# STEM: a tool for the analysis of short time series gene expression data

**DOI:** 10.1186/1471-2105-7-191

**Published:** 2006-04-05

**Authors:** Jason Ernst, Ziv Bar-Joseph

**Affiliations:** 1 Center for Automated and Learning and Discovery, School of Computer Science, Carnegie Mellon University, 5000 Forbes Ave., Pittsburgh, PA 15213, USA

## Abstract

**Background:**

Time series microarray experiments are widely used to study dynamical biological processes. Due to the cost of microarray experiments, and also in some cases the limited availability of biological material, about 80% of microarray time series experiments are short (3–8 time points). Previously short time series gene expression data has been mainly analyzed using more general gene expression analysis tools not designed for the unique challenges and opportunities inherent in short time series gene expression data.

**Results:**

We introduce the Short Time-series Expression Miner (STEM) the first software program specifically designed for the analysis of short time series microarray gene expression data. STEM implements unique methods to cluster, compare, and visualize such data. STEM also supports efficient and statistically rigorous biological interpretations of short time series data through its integration with the Gene Ontology.

**Conclusion:**

The unique algorithms STEM implements to cluster and compare short time series gene expression data combined with its visualization capabilities and integration with the Gene Ontology should make STEM useful in the analysis of data from a significant portion of all microarray studies. STEM is available for download for free to academic and non-profit users at .

## Background

**Figure 1:**
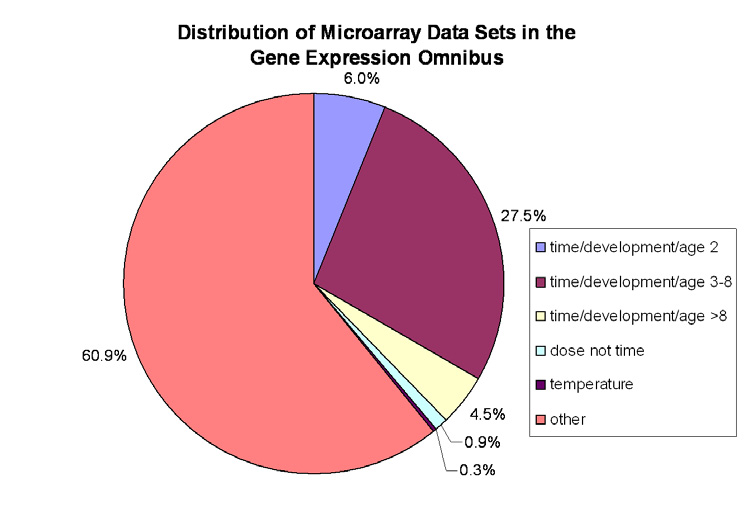
**Distribution of microarray experiments by type**. Summary of the 786 microarray datasets for human, mouse, rat, and yeast in the Gene Expression Omnibus as of August 2005. As can be seen, 27.5% of the sets are time series experiments with 3–8 time points. All of these sets were labeled as either time, development, or age in the database. An additional 1% percent contains other types of sequential experiments including dose or temperature response, with 3–8 different levels.

Microarray time series gene expression experiments are widely used to study a range of biological processes such as the cell cycle [1], development [2], and immune response [3]. Based on an analysis of the Gene Expression Omnibus [4], approximately a third of all microarray studies involve time series experiments with three or more time points, and of these time series experiments over 80% contain no more than eight time points (Figure 1). In many cases experimental costs prevent data from more time points from being collected. In some studies, particularly clinical studies, the availability of biological material can limit the number of time points collected. Thus, even if the price of microarray experiments were to go down short time series expression experiments would remain prevalent.

In this paper we introduce the Short Time-series Expression Miner (STEM), the first software application designed specifically for the analysis of short time series gene expression datasets (3–8 time points). Data from short time series gene expression experiments poses unique challenges. In these experiments thousands of genes are being profiled simultaneously while the number of time points is few. In such cases many genes will have the same expression pattern just by random chance. Furthermore as with any time series experiment, there are usually few, if any, full time series repeats from which to gain statistical power. STEM uses a method of analysis that takes advantage of the number of genes being large and the number of time points being few to identify statistically significant temporal expression profiles and the genes associated with these profiles [5]. STEM also supports Gene Ontology (GO) [6] enrichment analyses for sets of genes having the same temporal expression pattern providing the means for an efficient and statistically rigorous biological interpretation of significant temporal expression patterns. The integration of STEM with GO is bidirectional. STEM can easily determine and visualize the behavior of genes belonging to a given GO category, identifying which temporal expression profiles were enriched for genes in that category. Finally, STEM also supports the ability to compare temporal responses of genes across experimental conditions.

The novel clustering algorithm which STEM implements for short time series expression data is briefly reviewed in the Implementation section. For a detailed discussion of the clustering algorithm including experimental results on simulated data and a comparison with the *k*-means clustering algorithm on real biological data using GO we refer the reader to [5]. The main focus of this paper is on STEM's integration with GO, its support for comparing data sets across experimental conditions, its visualization capabilities, and a comparison with related software.

To date, researchers analyzing short time series expression data relied mainly on two types of software. The first is general gene expression analysis software implementing methods which do not take advantage of the sequential information in time series data. The second is gene expression time series analysis software implementing methods primarily designed for *longer *time series. General methods for gene expression analysis that are frequently applied to time series expression data include popular clustering methods such as hierarchical clustering [7], *k-*means clustering [8], and self-organizing maps [9]. These standard clustering methods ignore the temporal dependency among successive time points. Specifically, if we were to randomly permute the order of time points, the results of these methods would not change. Two software packages available for clustering time series gene expression that implement methods that take advantage of the temporal dependency of time points are the Graphical Query Language (GQL) [10] and the Cluster Analysis of Gene Expression Dynamics (CAGED) [11]. GQL implements a clustering algorithm based on a mixture of hidden markov models. CAGED implements a clustering algorithm based on autoregressive equations. Unlike STEM these methods generally require the estimation of many parameters and are thus less appropriate for short time series data. Also unlike STEM, both standard clustering methods and previously suggested temporal analysis methods do not differentiate between real and random patterns. This is a particular problem for short time series expression data since, as mentioned above, many genes may have the same expression pattern by random chance. A detailed comparison of STEM with the software implementing methods of analysis primarily designed for longer time series appears in the Discussion section of this paper.

STEM is freely available for download at [12] for non-commercial research purposes. A comprehensive and detailed manual is also available at [12] and as Additional file 1 to this paper.

## Implementation

STEM is implemented entirely in Java and will work with any operating system supporting Java 1.4 or later. Portions of the interface of STEM are implemented using a third party library, the Java Piccolo toolkit from the University of Maryland [13]. STEM also makes use of external Gene Ontology and gene annotation files. STEM can download these files directly from the websites of the Gene Ontology [14] or European Bioinformatics Institutes [15].

**Figure 2:**
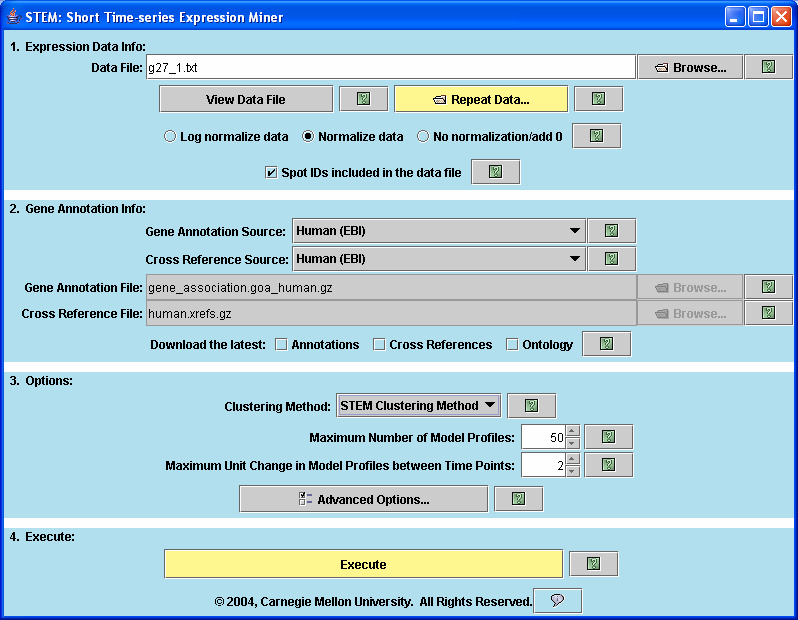
**STEM input interface**. The image shows the STEM input interface, which is divided into four sections. In the top section a user specifies the gene expression data and normalization options. In the second section a user specifies the gene annotation source, in this case the annotations are selected to be Human annotations from the European Bioinformatics Institute. In the third section a user specifies to either use the STEM clustering method or *k*-means, and can also change various parameter settings. The fourth section of the interface contains the execute button.

**Figure 3:**
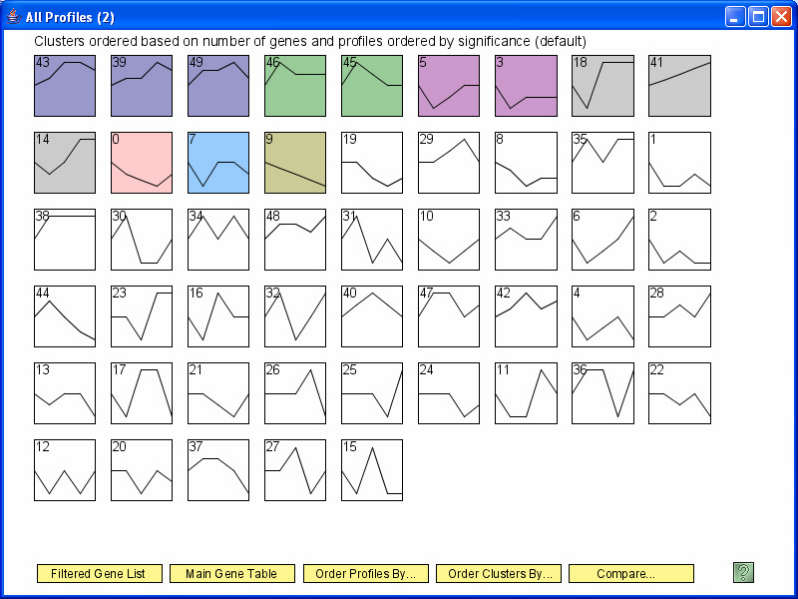
**Example model profiles overview interface**. The example data is drawn from an experiment measuring the response of gastric epithelial cells infected with the *vacA*^-^mutant strain of the pathogen *Helicobacter pylori *[3]. The data was sampled at five time points 0 h, .5 h, 3 h, 6 h, and 12 h. The data set was filtered to contain only the 2989 genes with no missing data (though STEM can handle missing data without filtering, see Additional file 1) that exhibited a .8 log base two fold increase or decrease for at least one time point. The number in the top left-hand corner of a profile box is the profile ID number. The colored profiles had a statistically significant number of genes assigned. Non-white profiles of the same color represent profiles grouped into a single cluster. By clicking on one of the buttons along the bottom of the window, a dialog window appears by which the profiles can be reordered by various criteria. Another button displays a table of all genes passing filter and the profile to which they were assigned. Clicking on a profile box brings up detailed information about the profile (Figure 5).

A user of STEM first specifies a tab delimited gene expression data file as input to STEM. Next, the user specifies a gene annotation source, and may adjust default parameters through the input interface shown in Figure 2. Following the input phase, the STEM clustering algorithm executes and a new window will appear displaying the clustering results (Figure 3). From this new window, a user will have the option to specify a comparison data set.

The novel clustering algorithm that STEM implements takes advantage of there being only a few time points in a dataset. The clustering algorithm first selects a set of distinct and representative temporal expression profiles (which we will refer to as model profiles from now on). These model profiles are selected independent of the data. The procedure for selecting the model profiles, and theoretical guarantees that the models profiles selected are representative and distinct appear in [5]. See Figure 3 for an example of a set of model profiles. The clustering algorithm then assigns each gene passing the filtering criteria (see Additional file 1 for details on gene filtering) to the model profile that most closely matches the gene's expression profile as determined by the correlation coefficient. Since the model profiles were selected independent of the data, the algorithm can then determine which profiles have a statistically significant higher number of genes assigned using a permutation test. This test determines an assignments of genes to model profiles using a large number of permutations of the time points (or columns). It then uses standard hypothesis testing to determine which model profiles have significantly more genes assigned under the true ordering of time points compared to the average number assigned to the model profile in the permutation runs. Significant model profiles can either be analyzed independently, or grouped together based on similarity to form clusters of significant profiles.

Based on a reviewer's suggestion, STEM now also provides an implementation of the *k*-means clustering algorithm. A user thus has the option to compare directly within STEM, results of STEM's novel clustering method with those produced using *k*-means. A user that still prefers the *k*-means clustering methodology for clustering short time series data, or is interested in using *k*-means to cluster other types of data for which the STEM clustering method does not apply, may still be interested in using STEM's implementation of *k*-means in order to leverage STEM's visualization capabilities and integration with GO. The results and discussion of STEM in this paper are presented using STEM's novel clustering method. For details on using the *k-*means clustering algorithm with STEM see Additional file 1.

## Results

### Model profiles overview interface

**Figure 4:**
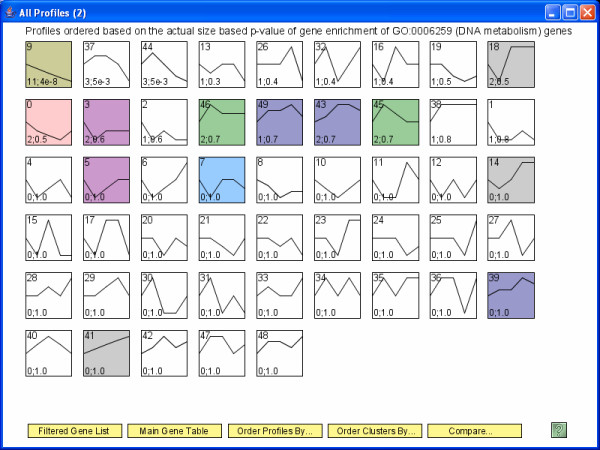
**Model profiles reordered interface**. The profiles from Figure 3 are reordered based on actual size based p-value enrichment for genes being annotated as belonging to the GO category DNA metabolism. For each profile the number of DNA metabolism genes assigned to it and the enrichment p-value appears in the lower left corner of the profile box.

A screenshot of the main interface window of STEM appears in Figure 3. In this window each box corresponds to one of the model temporal expression profiles. Clicking on a profile box displays a new window, described in the next subsection, with detailed information about the profile. The colored profiles have a statistically significant number of genes assigned. Colored profiles which have the same color are all similar to each other (based on correlation coefficients, see Additional file 1 for more details). These profiles are grouped together to form a cluster of significant profiles. By default profiles on the main window are ordered such that significant profiles appear before non-significant profiles, and among significant profiles those profiles of the same color appear next to each other. The profiles can be reordered based on the number of genes assigned, the number of genes expected, or their significance p-value. Additionally as we discuss below, the profiles can also be reordered based on their relevance to a given GO category (Figure 4), a user defined gene set, or profile(s) from a comparison experiment. When the profiles are reordered relevant information appears in the profile boxes.

The model overview screen is designed such that by default a user can visualize all profiles simultaneously, but as a result each profile box needs to be relatively small. At times however, a user will be interested in focusing on a small subset of neighboring profiles. The interface of STEM supports zooming and panning on any portion of the model profiles overview screen. The ability to zoom and pan is powered by the open source Java libraries of Piccolo [13].

### Model profile detailed information interface

**Figure 5:**
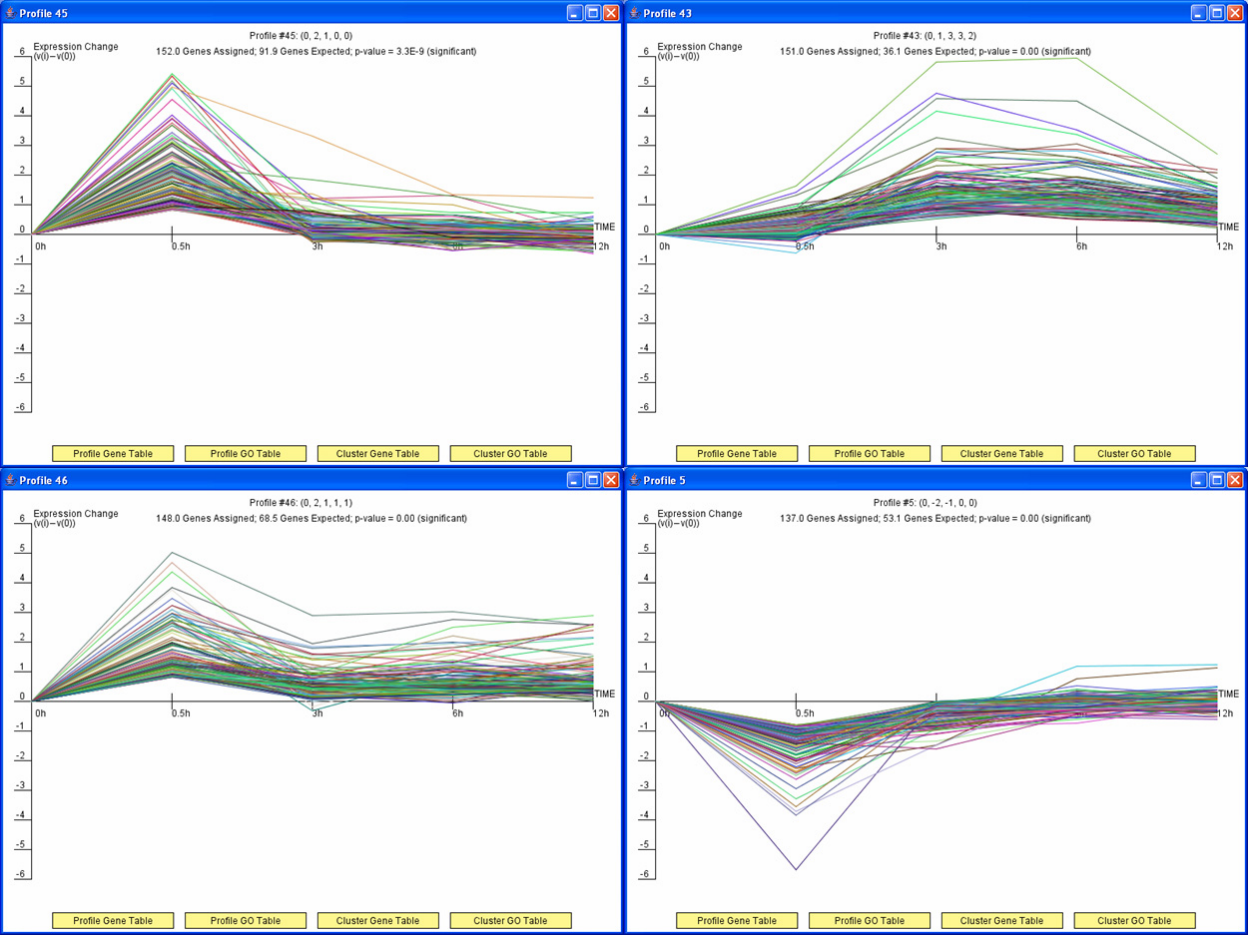
**Four model profile detailed information windows**. The above image are screenshots of four detailed model profile windows for the data of Figure 3. The four profiles shown were those which had the most genes assigned. All four profiles were significant, and the two profiles on the left were clustered together. Along the top of each window are statistics on the number of genes assigned to the profile, the number of genes expected, and the enrichment p-value. Along the bottom of the window is a button to display a table of genes assigned to the profile and a button to display a GO enrichment table for the profile (Figure 6). Additional buttons can be used to extract the cluster information for this profile.

**Figure 6:**
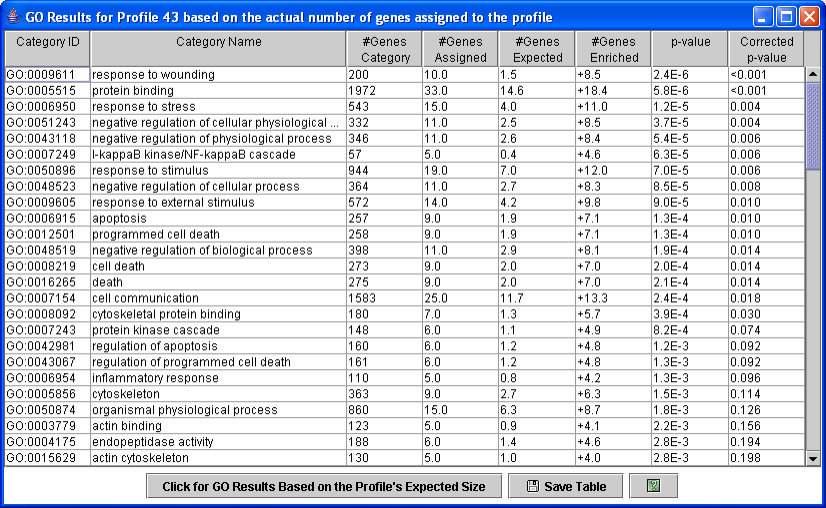
**Gene Ontology enrichment analysis table**. The image shows an example of a GO enrichment analysis table. This example table contains the GO enrichment results for the set of genes shown in the upper right of Figure 5 where the enrichment is computed based on actual size enrichment. The first two columns of the table are the GO category ID and name. The third column contains the total number of genes of each GO category on the microarray. The fourth column contains for each GO category total number of genes on the microarray that were also assigned to the profile. The fifth column contains the number of genes of that GO category that were expected to be assigned to the profile, in this case computed based on the profiles actual size. The sixth column contains how many more genes were assigned than expected. The seventh and eight columns contain the p-value and *corrected *p-values for the enrichment. Clicking on a row of the table brings up the list of genes of that GO category that were also assigned to the profile.

Clicking on a profile box on the model profiles overview interface displays a window with detailed information about the profile. Examples of such windows appear in Figure 5. The window contains a graph of the expression patterns for all the genes assigned to the profile, a count of the number of genes assigned, a count of the number of genes expected based on the permutation test, and the profile's p-value. The window also gives the option to display a table with all genes assigned to the profile, or to display a table for a GO enrichment analysis of the set of genes assigned to the profile (Figure 6). If the profile is a part of a non-singleton cluster of significant profiles, then there is also the option to display a table with a GO enrichment analysis for all cluster genes.

### Integration with the Gene Ontology

The Gene Ontology (GO) is a structured vocabulary for describing biological processes, cellular components, and molecular functions of gene products [6]. The ontology is a hierarchy of terms organized as a directed acyclic graph. GO term annotations of gene products is available for many organisms. A popular approach to gain biological insights from a set of identified genes of interest is to determine which GO terms annotations are overrepresented among the genes in the set. A number of software packages are available which can determine GO term enrichments in a set of genes (see [16] for a recent review). STEM's integration with GO allows a user to conduct gene enrichment analyses directly in STEM, avoiding the need for a user to export into a separate file each set of genes of interest and then import them into an external GO software. Additionally, STEM implements an expected size enrichment analysis not available in other software (see below). Also unique to STEM is its ability to allow a user with a GO category of interest to easily identify the significant temporal response patterns associated with this category.

The integration with GO is designed to be simple for the user, comprehensive, and current. A user can select from a drop down menu on the main interface any of 35 gene annotation sources available from the Gene Ontology [14] or European Bioinformatics Institutes websites [15]. STEM also accepts any user provided annotation file in the official 15 column gene annotation format, or a simpler two column annotation format. In fact there is no restriction that annotations be GO terms. The set of GO annotations used can be filtered based on evidence code, and restricted to specific subset of annotations (see Additional file 1 for more details). New versions of annotations and the ontology are frequently released, but STEM makes it easy for a user to keep these files up to date by simply having the user check an appropriate field when they want STEM to download the latest annotations or ontology.

#### Actual and expected size gene set enrichments

STEM implements two types of gene enrichments for a set of genes assigned to the same model temporal expression profile *r*. The default enrichment in STEM and the method used in other software is actual size based enrichment, in which the enrichment is computed using the hypergeometric distribution based on the number of genes in the set of interest. Formally denote by *N *the total number of unique genes on the microarray. Denote by *m *the total number of genes that are in the GO category of interest. Denote by *s*_*a *_the number of gene's assigned to profile *r*. Based on the hypergeometric distribution the p-value of seeing *v *or more genes in the intersection of the category of interest and profile *r *can be computed as:

∑i=vmin⁡(m,sa)(mi)(N−msa−i)(Nsa)
 MathType@MTEF@5@5@+=feaafiart1ev1aaatCvAUfKttLearuWrP9MDH5MBPbIqV92AaeXatLxBI9gBaebbnrfifHhDYfgasaacH8akY=wiFfYdH8Gipec8Eeeu0xXdbba9frFj0=OqFfea0dXdd9vqai=hGuQ8kuc9pgc9s8qqaq=dirpe0xb9q8qiLsFr0=vr0=vr0dc8meaabaqaciaacaGaaeqabaqabeGadaaakeaadaaeWbqaamaalaaabaWaaeWaaeaafaqabeGabaaabaGaemyBa0gabaGaemyAaKgaaaGaayjkaiaawMcaamaabmaabaqbaeqabiqaaaqaaiabd6eaojabgkHiTiabd2gaTbqaaiabdohaZnaaBaaaleaacqWGHbqyaeqaaOGaeyOeI0IaemyAaKgaaaGaayjkaiaawMcaaaqaamaabmaabaqbaeqabiqaaaqaaiabd6eaobqaaiabdohaZnaaBaaaleaacqWGHbqyaeqaaaaaaOGaayjkaiaawMcaaaaaaSqaaiabdMgaPjabg2da9iabdAha2bqaaiGbc2gaTjabcMgaPjabc6gaUnaabmaabaGaemyBa0MaeiilaWIaem4Cam3aaSbaaWqaaiabdggaHbqabaaaliaawIcacaGLPaaaa0GaeyyeIuoaaaa@51F6@

An advantage of the actual size enrichment is that it provides a means to externally validate a clustering algorithm, since the enrichment calculation makes no assumptions about how a set of genes was produced. Such a biological validation for the STEM clustering algorithm appears in [5].

Unlike other clustering algorithms, STEM's clustering algorithm also computes the expected number of genes matching a specific model profile. This leads to a new GO category enrichment p-value based on a profile's expected size. Formally, denote by *s*_*e *_the expected size of profile *r*. Then the p-value of seeing more than *v *genes belonging to both the category and profile *r *can be computed using the binomial distribution with parameters *m *and seN
 MathType@MTEF@5@5@+=feaafiart1ev1aaatCvAUfKttLearuWrP9MDH5MBPbIqV92AaeXatLxBI9gBaebbnrfifHhDYfgasaacH8akY=wiFfYdH8Gipec8Eeeu0xXdbba9frFj0=OqFfea0dXdd9vqai=hGuQ8kuc9pgc9s8qqaq=dirpe0xb9q8qiLsFr0=vr0=vr0dc8meaabaqaciaacaGaaeqabaqabeGadaaakeaadaWcaaqaaiabdohaZnaaBaaaleaacqWGLbqzaeqaaaGcbaGaemOta4eaaaaa@30D9@ as:

∑i=vm(mi)(seN)i(1−seN)N−i
 MathType@MTEF@5@5@+=feaafiart1ev1aaatCvAUfKttLearuWrP9MDH5MBPbIqV92AaeXatLxBI9gBaebbnrfifHhDYfgasaacH8akY=wiFfYdH8Gipec8Eeeu0xXdbba9frFj0=OqFfea0dXdd9vqai=hGuQ8kuc9pgc9s8qqaq=dirpe0xb9q8qiLsFr0=vr0=vr0dc8meaabaqaciaacaGaaeqabaqabeGadaaakeaadaaeWbqaamaabmaabaqbaeqabiqaaaqaaGqaciab=1gaTbqaaiab=LgaPbaaaiaawIcacaGLPaaaaSqaaiabdMgaPjabg2da9iabdAha2bqaaiabd2gaTbqdcqGHris5aOWaaeWaaeaadaWcaaqaaiabdohaZnaaBaaaleaacqWGLbqzaeqaaaGcbaGaemOta4eaaaGaayjkaiaawMcaamaaCaaaleqabaGaemyAaKgaaOWaaeWaaeaacqaIXaqmcqGHsisldaWcaaqaaiabdohaZnaaBaaaleaacqWGLbqzaeqaaaGcbaGaemOta4eaaaGaayjkaiaawMcaamaaCaaaleqabaGaemOta4KaeyOeI0IaemyAaKgaaaaa@4AFD@

An advantage of expected size enrichment occurs in the case in which the genes of multiple independent processes happen to have the same temporal expression pattern. In this case a temporal expression pattern could be very significant in terms of the number of genes assigned versus expected, but no GO category will appear enriched under an actual size enrichment test. However under an expected size enrichment test the GO categories could correctly be identified as being enriched. Expected size based enrichment is also useful for ordering temporal expression profiles to determine which are most relevant to a given GO category (see next subsection).

As many GO categories are being tested simultaneously, it is necessary to correct p-values using a multiple hypothesis correction. STEM can correct p-values using the Bonferonni correction, or in the case of actual size enrichment also by using a randomization test.

#### Bidirectional integration

STEM's integration with GO is bidirectional. In addition to allowing a user to determine for a given model profile what GO terms are significantly enriched, STEM can also determine for a given GO category what model profiles were most enriched for genes in that category. Given a GO category, STEM ranks the profiles based on their p-value enrichment for that category. The profiles on the main interface can be reordered based on either the actual or expected size enrichment. Figure 4 shows an image of the profiles of Figure 3 reordered by actual size based enrichment for the GO category DNA metabolism. In the bottom left hand corner of each profile box is the number of profile genes that belong to that GO category and the enrichment p-value. When the profiles are reordered by a GO category, upon opening the window with detailed information about the profile there is the option to plot just the subset of genes belonging to that GO category. STEM can also determine which cluster of significant profiles were most enriched for a GO category, and reorder the cluster of profiles according to the selected category.

### Comparing data sets across experimental conditions

Many microarray studies include a comparison of the temporal response of genes between experimental conditions. For example, researchers have compared the temporal response of genes infected with a wildtype pathogen to those infected with a knockout mutant version of the pathogen [3] or the response of genes when exposed to a certain chemical substance to their response when not exposed [17]. STEM supports the ability to compare expression data sets across experimental conditions even when only few time points are sampled (assuming that the number of time points are the same). STEM allows a user to investigate questions such as: "for a set of genes which had temporal response *X *in experiment *A*, what significant responses did they have in experiment *B*?". STEM uses the hypergeometric distribution to compute the significance of overlap between gene sets of model profiles of two experiments. Since the model profiles are defined independent of the data, the boundaries in expression space that they induce will remain the same between experiments. In contrast, cluster boundaries from traditional, data driven, clustering algorithms will change between experiments. STEM is thus able to detect significant sets of genes with the same expression profiles across experiments that might otherwise be missed if the clusters were defined differently across experiments. Furthermore since the model profiles in STEM are also selected to be distinct and representative of all expression profiles, STEM will determine for all pairs of distinct expression patterns if there is a significant gene set intersection. If the clusters had been formed with a data driven clustering algorithm no such guarantee is possible.

**Figure 7:**
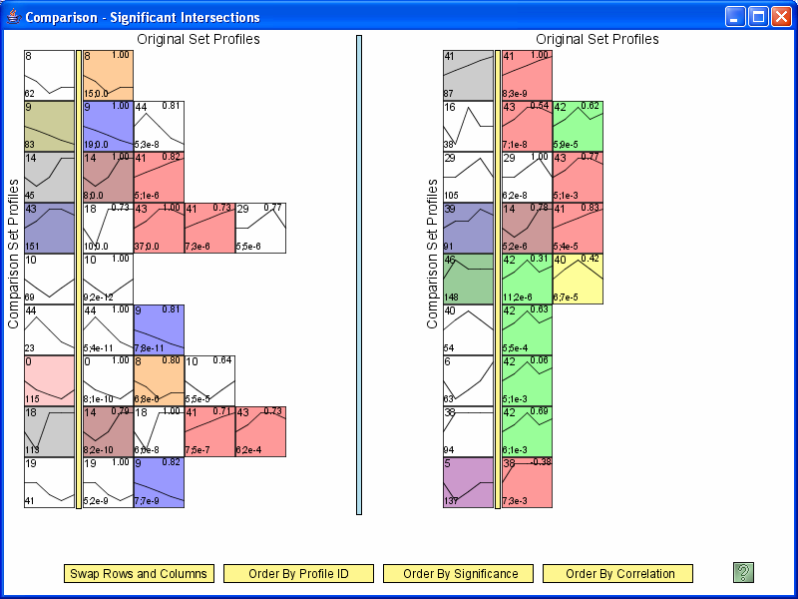
**Model profiles comparison interface**. The window above shows a portion of the comparison interface comparing the wildtype and *vacA*^- ^experiments from [3]. A profile to the immediate left of a yellow bar in this image is from the *vacA*^- ^experiment. A profile to the right of the yellow bar is from the wildtype experiment, and has a significant intersection (in terms of the genes assigned to them) with the profile to the left of the yellow bar in its row. The profile pairs are currently arranged based on the p-value of their intersection, with the temporal profile pairs that are most significant appearing to the top and left. The profile pairs can also be arranged based on their correlation or IDs.

Figure 7 shows a portion of the STEM interface which displays pairs of profiles from two experiments for which the gene set intersection is significant. The results are based on a comparison of an experiment measuring the response of gastric epithelial cells infected with the wildtype pathogen *Helicobacter pylori *to the response when infected with the *vacA*^- ^strain [3]. The window shows that many sets of genes had a consistent response across experiments. This result is consistent with the observation made in [3] that the phenotypical response of *vacA*^- ^infected cells was similar to the wildtype infected cells. The profile pairs on the comparison interface can be rearranged based on the significance of the intersection or how different the expression profiles are as measured by the correlation coefficient. On the main model profiles overview screen a user can reorder all the model profiles from one experiment based on the enrichment for a set of genes assigned to a profile or set of profiles in the other experiment.

## Discussion

A number of software packages implementing general methods for the analysis of gene expression data from multiple experiments have been used to analyze time series data. These include Cluster [7], EXPANDER [18], the MultiExperiment Viewer [19], and the High-Throughput Miner [20] among many others. Software packages using methods of analysis specifically designed for time series gene expression data are less common. Limited analysis functions designed for time series are available as a part of some broader software packages and also as stand alone scripts. For instance, detecting differentially expressed genes in time series data is available in Significance Analysis of Microarrays (SAM) [21] and Extraction of Differential Gene Expression (EDGE) [22], and detecting periodically expressed genes is a function in the GeneTS script [23]. TimeSearcher is an entire software application for visualizing time series data and has been applied to gene expression time series data [24], but it does not offer any automated analysis functions such as a clustering algorithm. The Gene Time Expression Warper [25] has support for aligning time series and also some visualization capabilities. ORIOGEN [26] implements a clustering algorithm designed for time series data when several full repeats are available, though having several full length time series repeats is not common. The two software packages most similar to STEM in the sense that they both support time series clustering and visualization without requiring repeats are the Cluster Analysis of Gene Expression Dynamics (CAGED) [11] and the Graphical Query Language (GQL) [10]. The clustering algorithm in CAGED is based on autoregressive equations, while the clustering algorithm in GQL is based on hidden markov models [27]. These methods either require estimating many parameters or using an over simplified model, and thus while useful for long time series are less appropriate for short time series data [5].

Unlike STEM, CAGED and GQL do not support comparing time series data sets. CAGED does not offer any GO analysis features, though it does have an automated report generation feature not available in STEM or GQL. GQL does provide support for determining GO enrichments for a cluster of genes. However, unlike STEM the support is not bidirectional, that is, there is no support for directly determining the temporal response of genes belonging to a GO category of interest. In terms of running time, STEM was the fastest when compared on the same real biological data. Table 1 summarizes the differences between STEM and CAGED and GQL.

## Conclusion

We have introduced, STEM, a new software package for analyzing short time series expression data. The software can find statistically significant patterns from short time series microarray experiments and can compare data sets across experiments. STEM presents its analysis of the data in a highly visual and interactive manner, and the integration with GO allows for efficient biological interpretations of the data. Through an analysis of the Gene Expression Omnibus we have estimated that short time series expression data is represented in about a quarter of all microarray studies. While STEM was designed with time series data in mind, it only makes the assumption that experiments can naturally be sequentially ordered. Thus, STEM could also be used for other types of sequential experiments such as dose response and temperature response experiments. The unique automated analysis capabilities of STEM combined with its visualization capabilities and integration with GO, should merit STEM to be a software of choice to analyze data from a significant portion of all microarray studies.

## Availability and requirements

**Project name: **STEM: Short Time-series Expression Miner

**Project home page: **

**Operating system(s): **Platform independent

**Programming language: **Java

**Other requirements: **Java 1.4 or higher

**License: **non-commercial research use license

**Any restrictions to use by non-academics: **license needed for commercial use

## Abbreviations

Cluster Analysis of Gene Expression Dynamics (CAGED)

Extraction of Differential Gene Expression (EDGE)

Gene Ontology (GO)

Graphical Query Language (GQL)

Short Time-series Expression Miner (STEM)

Significance Analysis of Microarrays (SAM)

## Authors' contributions

JE and ZBJ both contributed to the design of STEM. JE implemented STEM. Both JE and ZBJ participated in the drafting and revising of the manuscript, and read and approved the final manuscript.

## Supplementary Material

Additional File 1 **STEM user manual**. manual.pdf is a comprehensive user manual for STEM in pdf format. Click here for file

## Figures and Tables

**Table 1:** Comparison of time series microarray software packages. The table presents a comparison of three microarray time series analysis software packages: STEM, GQL, and CAGED. The execution time results are based on running on the same dataset as in Figure 3. The default settings of STEM and CAGED were used. For GQL the initial collection of HMMs was set to six two state HMMs and six three state HMMs. The experiments were carried out on a Pentium 1 GHz with 512 MB of RAM.

Software	Type of time series most appropriate for	Comparison Support	Cluster to GO Support	GO to Cluster Support	Automated Reports	Execution Time
STEM	short	Yes	Yes	Yes	No	17s
GQL	long	No	Yes	No	No	82s
CAGED	long	No	No	No	Yes	145s
