# Supplementary material for: STEM: a tool for the analysis of short time series gene expression data
Source: BMC Bioinformatics. 2006 Apr 5;7:191. doi: 10.1186/1471-2105-7-191 (PMC1456994; doi:10.1186/1471-2105-7-191)
Supplement: Additional File 1 — STEM user manual. manual.pdf is a comprehensive user manual for STEM in pdf format. [file 1471-2105-7-191-S1.pdf]

# STEM

## Short Time-series Expression Miner (v1.1)

*User Manual*

Jason Ernst (jernst@cs.cmu.edu)
Ziv Bar-Joseph
Center for Automated Learning and Discovery
School of Computer Science
Carnegie Mellon University

# Contents

**1 Introduction** **1**

1.1 STEM Clustering Method Overview . . . 1

1.2 Manual Overview . . . 1

**2 Preliminaries** **2**

**3 Input Interface** **2**

3.1 Expression Data Info . . . 2

3.2 Gene Annotation Info . . . 6

3.3 Options . . . 7

3.3.1 Filtering Options . . . 8

3.3.2 Model Profile Options . . . 9

3.3.3 Clustering Profiles Options . . . 10

3.3.4 Gene Annotations Options . . . 11

3.3.5 GO Analysis Options . . . 13

**4 Model Profiles Overview Interface** **14**

4.1 Main Gene Table . . . 15

4.2 Filtered Gene List . . . 16

4.3 Ordering Profiles . . . 17

4.4 Ordering Clusters of Profiles . . . 22

4.5 Zooming and Panning . . . 23

**5 Model Profile Details Interface** **23**

5.1 Gene Table . . . 27

5.2 Gene Enrichment Analysis Table . . . 27

**6 Comparison** **30**

**7 K-means** **35**

**A Defaults File Format** **40**

**B Using STEM for Standard Gene Ontology Enrichment Analysis** **42**

**C Gene Annotation Sources** **43**

# 1 Introduction

Welcome to STEM!

STEM is an acronym for the Short Time-series Expression Miner, a software program designed for clustering, comparing, and visualizing gene expression data from short time series microarray experiments (~8 time points or fewer). STEM implements a novel method for clustering short time series expression data that can differentiate between real and random patterns. STEM is also integrated with the Gene Ontology (GO) [4] allowing efficient biological interpretations of the data.

## 1.1 STEM Clustering Method Overview

The novel clustering method that STEM implements first defines a set of distinct and representative model temporal expression profiles independent of the data. These model profiles correspond to possible profiles of a gene's change in expression over time. The model profiles all start at 0, and then between two time points a model profile can either hold steady or increase or decrease an integral number of time units up to a parameter value. Gene expression times series are transformed to start at 0, and each gene is assigned to the model profile to which its time series most closely matches based on the correlation coefficient. The number of genes assigned to each model profile is then computed. The number of genes expected to be assigned to a profile is estimated by randomly permuting the original time point values, renormalizing the gene's expression values, then assigning genes to their most closely matching model profiles, and repeating for a large number of permutations. The average number of genes assigned to a model profile over all permutations is used as the estimate of the expected number of genes assigned to the profile. The statistical significance of the number of genes assigned to each profile versus the number expected is also then computed. Statistically significant model profiles which are similar to each other can be grouped together to form clusters of profiles. The biological significance of the set of genes assigned to the same profile or the same cluster of profiles can then be assessed using a GO enrichment analysis. For a more detailed discussion of the novel method STEM uses to cluster genes and associate statistical significance with genes having the same expression profile see [3].

## 1.2 Manual Overview

The remainder of the main portion of the manual contains six sections. Section 2 contains instructions on installing and starting STEM. Section 3 discusses the input to STEM including execution options and data file formats. Section 4 describes the model profile overview interface, which allows a user to visualize on a zoomable interface a large number of model profiles and order them based on their relevance to a GO category or user defined gene set. Section 5 describes the interface for obtaining detail information about a model profile or cluster of profiles including a table of genes assigned and a table of GO category enrichments. Section 6 describes STEM features to compare two data sets from different experimental conditions. STEM also provides an implementation of the standard $K$-means clustering algorithms which is described in Section 7. Sections 3-6 are presented assuming a user is interested in the novel STEM clustering method. Using $K$-means in STEM is similar, and the differences are discussed in Section 7. Most, but not all of the information, contained in this manual can also be obtained by clicking on the help icons throughout the software.

## 2 Preliminaries

- To use STEM a version of Java 1.4 or later must be installed. If Java 1.4 or later is not currently installed, then it can be downloaded from **http://www.java.com**.
- To install STEM simply save the file **stem.zip** locally and then unzip it. This will create a directory called **stem**.
- To execute STEM in Windows with its default initialization options simply double click on the file **stem.cmd** in the **stem** directory.
- To execute STEM from a command line change to the **stem** directory type and then type:

  ```
  java -mx1024M -ms512M -jar stem.jar
  ```

  If Java gives an error message indicating that there is not enough memory on the computer available to start STEM, then remove the -ms512M option. For slightly better time performance at the cost of more memory usage, replace the -ms512M option with -ms1024M.
- STEM can be started with its initial settings specified in a default settings file. The format of a default setting file is specified in Appendix A. To have STEM load its initial settings from a default settings file, from the command line append -d followed by the name of the default settings file to the above command. For instance to have STEM start with the settings specified in the file **defaults.txt** use the command:

  ```
  java -mx1024M -ms512M -jar stem.jar -d defaults.txt
  ```

## 3 Input Interface

The first window that appears after STEM is launched is the input interface (Figure 1). The interface is divided into four sections. In the top section a user specifies the expression data files and normalization options for the data. In the second section a user specifies the gene annotation information. In the third section a user specifies the desired clustering algorithm and various execution options. These three sections of the interface are described in more detail in the next three subsections. In the fourth section of the interface there is a button which when pressed causes STEM to execute the selected clustering algorithm, and then display the output interface described in Section 4. If the data file does not have two or more time points then results for a standard gene enrichment analysis will be displayed. For details about using STEM for standard gene enrichment analysis on non-time series data consult Appendix B.

### 3.1 Expression Data Info

The first field in the expression data section of the interface is the *Data File* field where a user specifies the input data file. An input data file consists of gene symbols, time series expression values, and optionally spot IDs. Spot IDs uniquely identify an entry in the data file, and if they are not included in the data file, then they will be automatically generated. While spot IDs must be unique, the same gene symbol may appear multiple times in

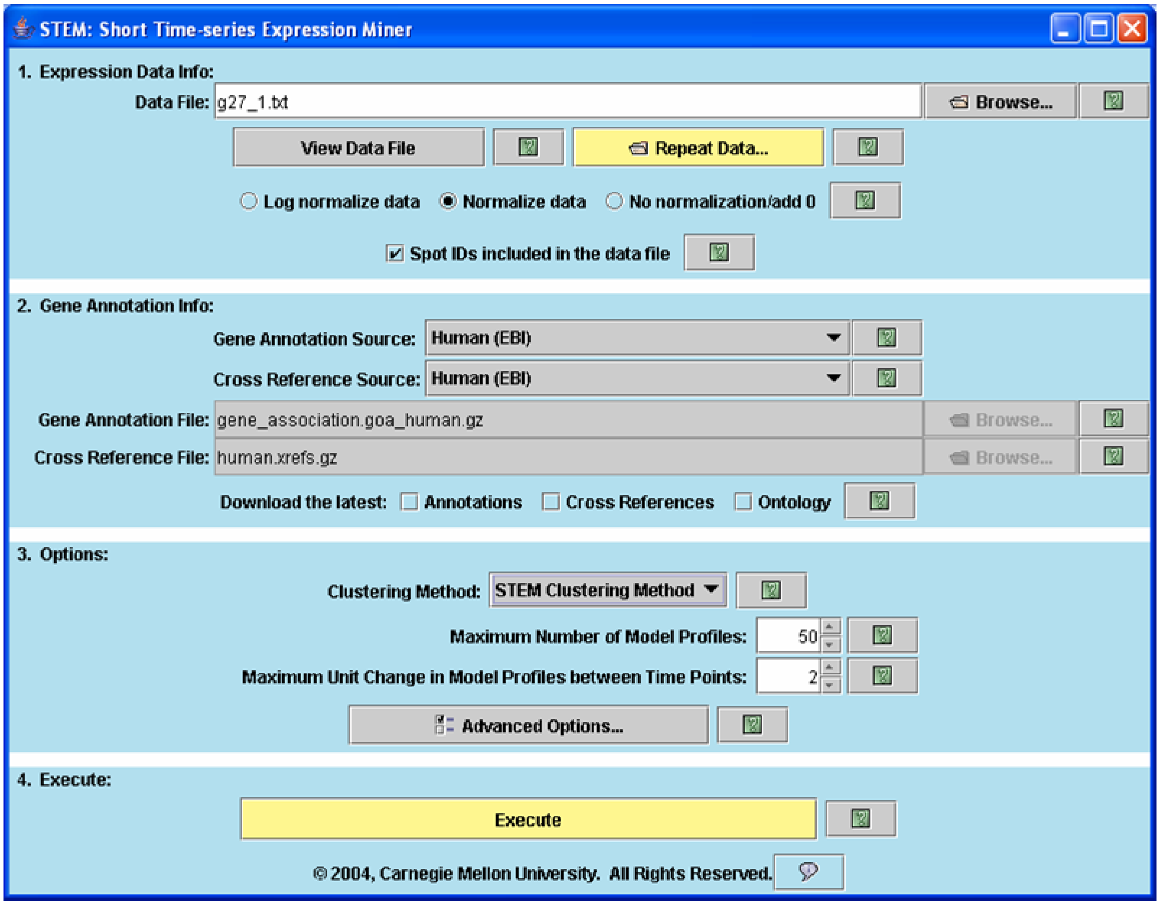


Figure 1: Above is the main input interface, which is the first screen that appears when STEM is launched. From this screen a user specifies the input data, gene annotation information, and various execution options. Pressing the execute button at the bottom of the interface causes the clustering and gene enrichment analysis algorithms to execute and then a new interface, described in Section 4, to appear.

the data file corresponding to the same gene appearing on multiple spots on the array. Expression values for the same gene will be averaged using the median before further analysis on the data is conducted.

A sample data file as it would appear in Microsoft Excel is shown in Figure 2. The first column, which appears in yellow, is optional, and if included contains spot IDs. If the data file includes the spot IDs column, then the field *Spot IDs included in the data file* on the input interface must be checked, otherwise the field must be unchecked. The next column, or the first column if spot IDs are not included in the data file, contain gene symbols. If a gene symbol is not available then the field can be left empty or a '0' can be placed in it. Both the spot ID field and the gene symbol field may contain multiple entries delimited by a semicolon (';'), pipe ('|'), or comma (','). The sub-entries in the field are only relevant in the context of gene annotations described in the next section. The remaining columns contain the expression value at each time point ordered sequentially based on time. If an expression value is missing, then the field should be left empty.

The first row of the data file contains column headers, and each row below the column header corresponds to a spot on the microarray. Each column must be delimited by a tab. The tab-delimited input data file should be

| | A | B | C | D | E | F | G |
|---|---|---|---|---|---|---|---|
| 1 | SPOT | Gene Symbol | 0h | 0.5h | 3h | 6h | 12h |
| 2 | 1 | ZFX | -0.027 | 0.158 | 0.169 | 0.193 | -0.165 |
| 3 | 2 | ZNF133 | 0.183 | -0.068 | -0.134 | -0.252 | 0.177 |
| 4 | 3 | USP2 | -0.67 | -0.709 | -0.347 | -0.779 | -0.403 |
| 5 | 4 | DSCR1L1 | -0.923 | -0.51 | -0.718 | -0.512 | -0.668 |
| 6 | 5 | WNT5A | -0.471 | -0.264 | -0.269 | -0.154 | -0.254 |
| 7 | 6 | VHL | -0.327 | -0.378 | -0.229 | -0.264 | -0.072 |
| 8 | 7 | TCF3 | -0.021 | 0.129 | -0.209 | -0.245 | 0.036 |
| 9 | 8 | TCN2 | -0.492 | -0.41 | -0.306 | -0.494 | -0.273 |
| 10 | 9 | TIMP1 | -0.111 | 0.351 | 0.168 | 0.129 | -0.293 |
| 11 | 10 | SERPINA7 | -0.468 | -0.488 | -0.199 | -0.144 | -0.185 |

Figure 2: Above is a sample input data file when viewed in Microsoft Excel. The first column, shown in yellow, contains spot IDs and is optional. If the column is included then the field *Spot IDs included in the data file* on the input interface must be checked, otherwise the field must be unchecked and the first column contain gene symbols. The columns containing the time series of gene expression values come after the gene symbol column. The sample data in this figure and throughout the manual comes from [5]

g27_1.txt

| SPOT | Gene Symbol | 0h | 0.5h | 3h | 6h | 12h |
|---|---|---|---|---|---|---|
| 1 | ZFX | -0.027 | 0.158 | 0.169 | 0.193 | -0.165 |
| 2 | ZNF133 | 0.183 | -0.068 | -0.134 | -0.252 | 0.177 |
| 3 | USP2 | -0.67 | -0.709 | -0.347 | -0.779 | -0.403 |
| 4 | DSCR1L1 | -0.923 | -0.51 | -0.718 | -0.512 | -0.668 |
| 5 | WNT5A | -0.471 | -0.264 | -0.269 | -0.154 | -0.254 |
| 6 | VHL | -0.327 | -0.378 | -0.229 | -0.264 | -0.072 |
| 7 | TCF3 | -0.021 | 0.129 | -0.209 | -0.245 | 0.036 |
| 8 | TCN2 | -0.492 | -0.41 | -0.306 | -0.494 | -0.273 |
| 9 | TIMP1 | -0.111 | 0.351 | 0.168 | 0.129 | -0.293 |
| 10 | SERPINA7 | -0.468 | -0.488 | -0.199 | -0.144 | -0.185 |

Figure 3: A sample input data file displayed in a table after the button *View Data File* on the input interface was pressed.

an ASCII text file or a GNU zip file of an ASCII text file. A tab-delimited text file can easily be generated in Microsoft Excel by choosing *Text(Tab delimited)* as the *Save as type* type under the *Save As* menu. To view the contents of the data file from the interface press the button *View Data File* and then a table such as in Figure 3 will appear.

Before gene expression time series are matched against model temporal expression profiles, the time series must be transformed to start at 0. The transformation that is used to do this can be selected to be of one of three types: *Log normalize data*, *Normalize data*, or *No normalization/add 0*. Given a time series vector of gene expression values $(v_0, v_1, v_2, ..., v_n)$ the transformations are as follows:

- *Log normalize data* – transforms the vector to $(0, \log_2(\frac{v_1}{v_0}), \log_2(\frac{v_2}{v_0}), ..., \log_2(\frac{v_n}{v_0}))$
- *Normalize data* – transforms the vector to $(0, v_1 - v_0, v_2 - v_0, ..., v_n - v_0)$
- *No normalization/add 0* – transforms the vector to $(0, v_0, v_1, v_2, ..., v_n)$

It is recommended that after transformation a time series represent the log ratios of the gene expression levels versus the level at time point 0. Time point 0 usually corresponds to a control before the experimental conditions were applied. If the input data file contains raw expression values as from an Oligonucleotide array, then the *Log normalize data* option should be selected. If any values are 0 or negative and the *Log normalize data* option is selected, then these values will be treated as missing. If the input data file already represents the log ratio of a sample against a control as is often the case when the data is from a two channel cDNA array and an experiment was conducted at time point 0, then the *Normalize data* option should be selected. In this case after normalization the transformed values will represent the log change ratio versus time point 0. If the input data file already contains log ratio data against a control, but no time point 0 experiment was conducted, then the *No normalization/add 0* option should be selected. In this case the assumption is made that had a time point 0 experiment been conducted the expression level in both channels would have been equal.

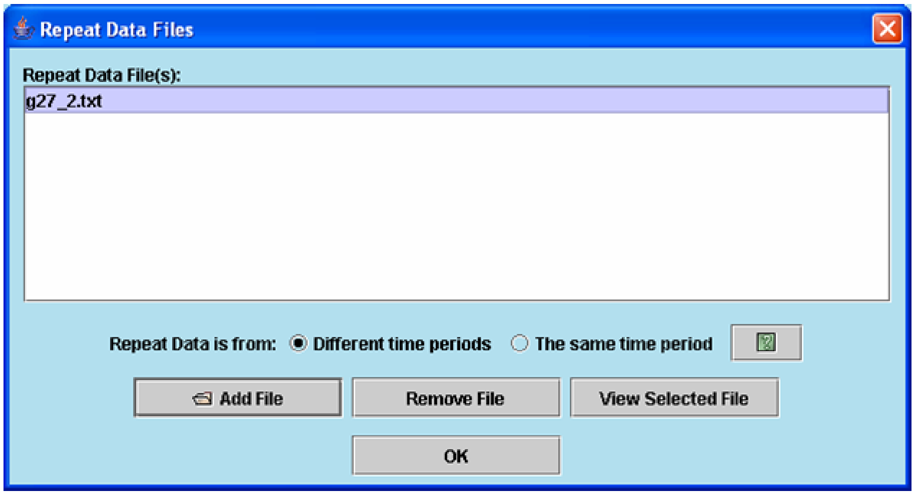


Figure 4: The above window is used to specify repeat data files. A user can add or remove repeat files with the *Add File* and *Remove File* buttons. A user also needs to specify whether the repeat data samples are from the same time period or different time periods as the original data. The contents of a repeat file can be viewed by selecting the repeat file and then pressing the *View Selected File* button.

Pressing the *Repeat Data* button brings up an interface as shown in Figure 4. The *Repeat Data* button on the main input interface is yellow if there is currently one or more repeat data files specified, otherwise it is gray. Repeat data files must have the same format as the original data file, including the same number of rows and columns. Repeat data values will be averaged with the values from the original data file using the median.

Repeat data can be selected to be from either *Different time periods* or *The same time period*. If the data is from *Different time periods* then data was collected over multiple distinct time series, but presumably at the same sampling rate. If the data is from *The same time period* then this implies multiple measurements were collected at each time point during one time series. If the repeat data is selected to be from the *The same time period*, then the file to which any two column of values for the same time point could be interchanged without effect, while if the repeat data is selected to be from *Different time periods* this is not the case. If the repeat data is from *Different time periods*, the repeat data will be averaged after normalization, while if the repeat data is from *The Same Time Period* the repeat data will be averaged before normalization. In the case the repeat data

is from *Different time periods*, the repeat data can be used to filter genes with inconsistent expression patterns and also to provide noise estimates by which to base clustering model profiles as explained in Section 3.3.

## 3.2 Gene Annotation Info

| | A | B |
|---|---|---|
| 1 | SEPW1 | GO:0016491;GO:0000004;GO:0008372 |
| 2 | PRPF8 | GO:0008248;GO:0006397;GO:0005634;GO:0005682 |
| 3 | PRPF4 | GO:0008248;GO:0000398;GO:0008380;GO:0005681 |
| 4 | JMJD2B | GO:0003677;GO:0006355 |
| 5 | JMJD2A | GO:0003677;GO:0006355 |
| 6 | AOX1 | GO:0004031;GO:0004854;GO:0005489;GO:0016491;GO:0030151;GO:0006118;GO:0006800;GO:0006954 |
| 7 | OBP2B | GO:0005215;GO:0005549;GO:0000004;GO:0006810;GO:0007608;GO:0007635;GO:0008372 |
| 8 | OBP2A | GO:0005215;GO:0005549;GO:0000004;GO:0006810;GO:0007608;GO:0008372 |
| 9 | PNLIP | GO:0004806;GO:0016787;GO:0006641;GO:0016042 |
| 10 | STK6 | GO:0004674;GO:0005524;GO:0016740;GO:0006468;GO:0007049;GO:0007067;GO:0005634;GO:0005819 |

Figure 5: Annotation file in a two column format. The first column contains gene symbols or spot IDs while the second column contains category IDs. Annotation files can also be in the official 15 column format.

In the second section of the interface a user specifies the gene annotation information. Both gene symbols or spot IDs can be annotated as belonging to an official Gene Ontology (GO) category or a user defined category. If a gene is annotated as belonging to an official category in the Gene Ontology, then it will automatically also be annotated as belonging to any ancestor category in the ontology hierarchy. The first field in this section of the interface is the *Gene Annotation Source*. This field can be set to either *User provided*, *No annotations*, or one of 35 annotation data sets provided by Gene Ontology Consortium members. A full list of the 35 data sets can be found in Appendix C. More information about these annotation sets can be found at `http://www.geneontology.org/GO.current.annotations.shtml`, and for the annotation sets provided by the European Bioninformatics Institute (EBI) also at `http://www.ebi.ac.uk/GOA/`. One of the 35 data sets is the EBI UniProt set. Subsets of this data set with annotations specific to a large number of organisms can be found at `http://www.ebi.ac.uk/GOA/proteomes.html` and are not included in the list of 35 data sets. If one of the 35 data sets is selected, then the annotation file corresponding to the source will appear in the *Gene Annotation File* text box uneditable. If *User provided* is selected, then the *Gene Annotation File* text box will become editable, and a user can specify a gene annotation file. Selecting *No annotations* is equivalent to selecting *User Provided* and leaving the field empty.

A gene annotation file can be in one of two formats:

1. The gene annotation file can be in the official 15 column gene annotation file format described at `http://www.geneontology.org/GO.annotation.shtml#file`. All 35 of the data sets provided by Gene Ontology Consortium members are in this format. If the file is in this format any entry in the columns *DB_Object_ID* (Column 2), *DB_Object_Symbol* (Column 3), *DB_Object_Name* (Column 10), or *DB_Object_Synonym* (Column 11) will be annotated as belonging to the GO category specified in Column 5 of the row. If the entry in the *DB_Object_Symbol* contains an underscore ('_'), then the portion of the entry before the underscore will also be annotated as belonging to the GO category since under some naming conventions the portion after the underscore is a symbol for the database that is not specific to the gene. The *DB_Object_Synonym* column may have multiple symbols delimited by either a semicolon (';'), comma (','), or a pipe ('|') symbol and all will be

annotated as belonging to the GO category in Column 5. Note that the exact content of the *DB_Object_ID*, *DB_Object_Symbol*, *DB_Object_Name*, and *DB_Object_Synonym* varies between annotation source, consult the README files available at `http://www.geneontology.org/GO.current.annotations.shtml` to find out more information about the content of these fields for a specific annotation source.

2. The alternative format for an annotation file is two columns delimited by a tab as illustrated in Figure 5. The first column contains gene symbols or spot IDs and the second column contains category IDs. The entries in each column are delimited by a semicolon (';'), comma (','), or a pipe ('|') symbol. If the same gene symbol or spot ID appears on multiple rows, then the union of all its annotations is used.

Matches between gene symbols in the data file and the annotation file is not case sensitive. Gene annotation files can either be in an ASCII text format or a GNU zip file of an ASCII text file.

Below the *Gene Annotation Source* field, is the *Cross Reference Source* field which controls the entry in the *Cross Reference File* field. Cross references are useful in the case that the naming convention used for genes in the data file is different than what is used in the gene annotation file. A cross reference file establishes that two or more symbols refer to the same gene. Note that the cross references is only used to map between gene symbols, and not spot IDs and gene symbols. The *Cross Reference Source* field gives the option to select either *User Provided*, *No cross references*, or cross references for *Arabidopsis*, *Chicken*, *Cow*, *Human*, *Mouse*, *Rat*, or *Zebrafish* provided by the European Bioinformatics Institute (EBI). If *User Provided* is selected for the cross reference file field, then the *Cross Reference File* field becomes editable, and a user can specify a cross reference file. Any gene symbols listed on the same line in the cross reference file will be considered equivalent. The symbols on a line can be delimited by either a tab, semicolon (';'), comma (','), or a pipe ('|'). As with gene annotations files a cross reference file can either be in an ASCII text file or GNU zip version of an ASCII text file.

At the bottom of the gene annotation section of the interface is the phrase *Download the latest* and then three checkboxes, *Annotations*, *Cross References*, and *Ontology*. If the *Annotations* box is checked, then the file listed in the *Gene Annotation File* box will be downloaded from `ftp://ftp.geneontology.org/go/gene-associations/` unless it is an EBI data source in which case it will be downloaded from `ftp://ftp.ebi.ac.uk/pub/databases/GO/goa/`. If the *Cross References* box is checked, then the file listed in the *Cross Reference File* box will be downloaded from `ftp://ftp.ebi.ac.uk/pub/databases/GO/goa/`. If the *Ontology* field is checked, then the file **gene_ontology.obo** will be downloaded from `http://www.geneontology.org/ontology/gene_ontology.obo`. If the annotation, cross reference, or ontology file is required for use, and not present in the **stem** directory, then the corresponding field will be checked and there will not be an option to uncheck the field forcing download of the file(s). If the *Gene Annotation Source* is set to *User Provided* then there will not be an option to download the gene annotation file, and likewise for the cross reference source field and cross reference file. Upon pressing the execute button, the files corresponding to the checked fields will be downloaded.

## 3.3 Options

In the third section of the interface a user has the option to specify a variety of execution options for STEM. The first option a user specifies is the *Clustering Method* which can be set to either *STEM Clustering Method* or *K-means*. The STEM clustering method is the novel clustering method STEM implements specifically designed for short time series expression data briefly described in Section 1.1 and described in more detail in [3]. STEM's

implementation of the *K-means* algorithm is discussed in Section 7. Assuming the user selects the *STEM Clustering Method*, then two options related to selecting temporal model expression profiles appear directly on the main input interface window. These options are:

- *Maximum Number of Model Profiles* – This parameter specifies the maximum number of model profiles that can be selected. Model profiles are selected from a larger set of candidate model profiles. Candidate model profiles are non-constant profiles which start at 0 and increase or decrease an integral number of units that is less than or equal to the value of the *Maximum Unit Change in Model Profiles between Time Points*. See [3] for a discussion on how a set of distinct and representative set of model profiles are selected from the larger set of candidate model profiles. If the value of *Maximum Number of Model Profiles* is set to 0, then there is no hard upper bound on the number of model profiles, and the number of model profiles is limited only by the number of candidate profiles and the *Maximum Correlation* parameter under the *Model Profiles* section of *Advanced Options*.
- *Maximum Unit Change in Model Profiles between Time Points* – This parameter specifies the maximum number of a units a model profile may change between time points. A model profile between two consecutive time points can either stay constant, or increase or decrease an integral number of units up to this parameter value.

If a user selects $K$-means clustering then these two options do not appear and instead two options specific to $K$-means clustering appear (see Section 7). The remaining options can be accessed by pressing the *Advanced Options* button. These remaining options are divided into five panels, *Filtering* (Figure 6), *Model Profiles* (Figure 7), *Clustering Profiles* (Figure 8), *Gene Annotations* (Figure 9), and *GO Analysis* (Figure 10), which are discussed in the next subsections.

### 3.3.1 Filtering Options

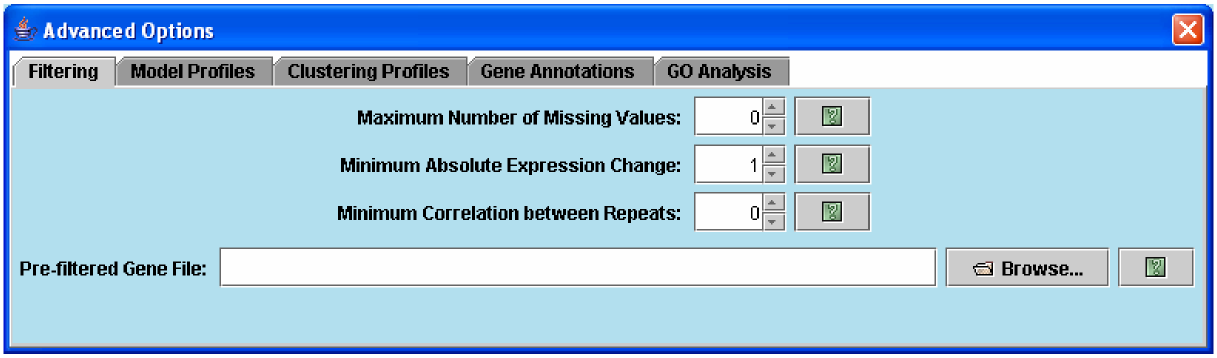


Figure 6: The above panel is used to specify gene filtering options.

Through the parameters on the Filtering panel shown in Figure 6 a user can adjust the criteria STEM uses to filter genes. If a gene is filtered, then it will be excluded from further analysis. Genes can be filtered if they do not show a sufficient response to experimental conditions (*Minimum Absolute Expression Change*), there are too many missing values (*Maximum Number of Missing Values*), or the gene expression pattern over repeats is too

inconsistent (*Minimum Correlation between Repeats*). If the *Log normalize data* or *Normalize data* options are selected, a gene will automatically be filtered if its expression value at the first time point is missing. A user can also filter genes by criteria not implemented in STEM, in which case a *Pre-filtered Gene File* should be specified. Below is a more detailed description of the parameters on the filtering panel:

- *Maximum Number of Missing Values* – A gene will be filtered if the number of missing values exceeds this parameter.

- *Minimum Absolute Expression Change* – A gene will be filtered if the absolute value of its expression at every time point after the selected transformation is applied (*Log normalize data*, *Normalize data*, or *No normalization/add 0*) is below this value.

- *Minimum Correlation between Repeats* – This parameter controls filtering of genes which do not display a consistent temporal profile across repeat experiments and only applies if there is repeat data selected to be from *Different time periods*. If there is a single repeat file, a gene will be filtered if its correlation between the original data set and the repeat set is below this parameter. If multiple repeats are available, then the gene will be filtered if the median of all its pairwise correlations between experiments is below this parameter.

- *Pre-filtered Gene File* – This file is optional. If included any genes listed in the file will be considered part of the initial base set of genes during a Gene Ontology (GO) enrichment analysis in addition to any genes included in the data file. Using this file thus allows one to filter genes from the data by a criteria not implemented in STEM by excluding them from the data file, but still include the filtered genes as part of the base set of genes during a GO enrichment analysis. If genes appear in both *Pre-filtered Gene File* file and the data file, then the gene will only be added to the base set once. The format of this file is the same as a data file, except including the time series expression values is optional and if included they will be ignored. As with a data file if the field *Spot IDs included in the data file* is checked, then the first column will contain spot IDs and the second column will contain gene symbols, otherwise the first column will contain gene symbols.

### 3.3.2 Model Profile Options

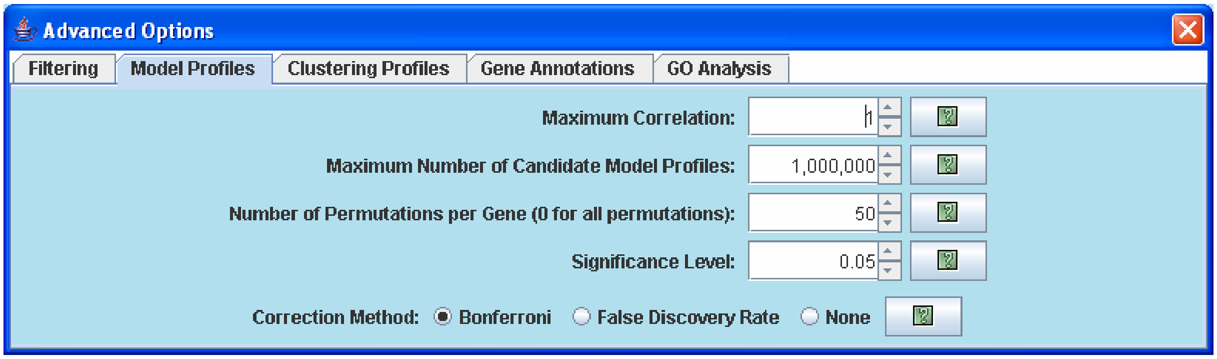


Figure 7: The above panel is used to specify options for selecting model profiles and assessing their statistical significance.

The panel used to adjust parameters related to model profiles appears in Figure 7. The parameters on this panel are only relevant to the STEM clustering method and not $K$-means clustering. The first two parameters *Maximum Correlation* and *Maximum Number of Candidate Model Profiles* influence the selection of model profiles along with the two parameters from the main input interface, *Maximum Number of Model Profiles* and *Maximum Unit Change in Model Profiles between Time Points.* The final three parameters, *Number of Permutations per Gene*, *Significance Level*, and *Correction Method* are related to the statistical test of whether a profile has a statistically significant number of genes assigned. The parameters on this panel are described below:

- *Maximum Correlation* – This parameter specifies the value that the maximum correlation between any two model profiles must be below, and thus can be used to guarantee that two very similar profiles will not be selected. Lowering this parameter could have the effect that the number of model profiles selected is less than the *Maximum Number of Model Profiles* even if more candidate model profiles are available. This parameter's maximum value is 1, thus preventing two perfectly correlated model profiles from being selected.

- *Maximum Number of Candidate Model Profiles* – Candidate model profiles are non-constant profiles which start at 0 and increase or decrease an integral number of units that is less than or equal to the value of the *Maximum Unit Change in Model Profiles between Time Points.* If the number of candidate model profiles exceeds this parameter, then instead of explicitly generating all candidate model profiles a subset of candidate model profiles of this size will be randomly selected. In most cases there will be no need to adjust this parameter.

- *Number of Permutations per Gene* – This parameter specifies the number of permutations of time points that should be randomly selected for each gene when computing the expected number of genes assigned to each of the model profiles. If this parameter is 0, then all permutations are used. Increasing the number of permutations will lead to slightly greater accuracy at the expense of greater execution time.

- *Significance Level* – The significance level at which the number of genes assigned to a model profile as compared to the expected number of genes assigned should be considered significant. If the *Correction Method* parameter for multiple hypothesis testing is *Bonferroni*, then this parameter is the significance level before applying a Bonferroni correction. If *Correction Method* is *False Discovery Rate*, then this parameter is the false discovery rate. If *Correction Method* is *none*, then this parameter is the uncorrected significance level.

- *Correction Method* – The significance level can be corrected for the fact that multiple profiles are being tested for significance. The correction can be a *Bonferroni* correction where the significance level is divided by the number of model profiles or the less conservative *False Discovery Rate* control [2]. If *none* is selected then no correction is made for the multiple significance tests. Note that this parameter for multiple test correction for model profiles is unrelated to the corrected p-values in a GO enrichment analysis.

### 3.3.3 Clustering Profiles Options

The two parameters on the clustering profile panel, shown in Figure 8, control the grouping of significant model profiles into clusters. The parameters on this panel are again only relevant to the STEM clustering method and

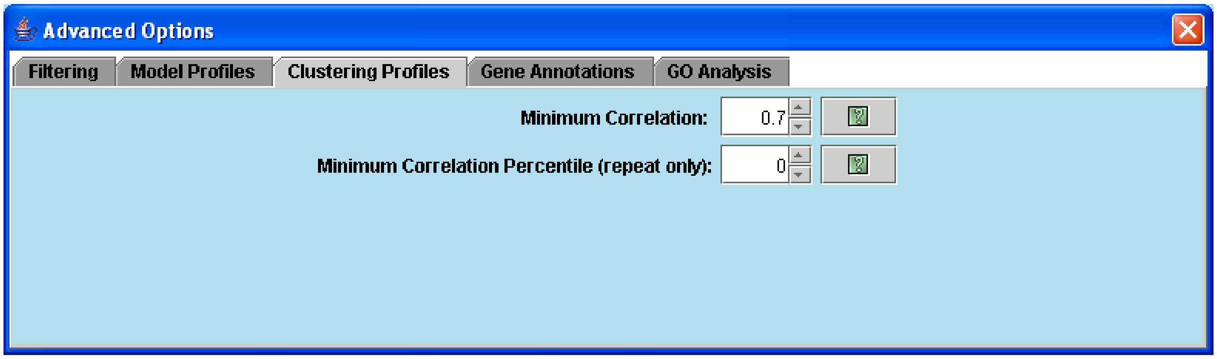


Figure 8: The above panel is used to specify options for grouping statistically significant model profiles.

not $K$-means clustering. The parameters control how similar two model profiles must be if they are grouped together. The two parameters are as follows:

- *Minimum Correlation* – Any two model profiles assigned to the same cluster of profiles must have a correlation above this parameter's value. Increasing this value will lead to more clusters with fewer model profiles per cluster, while decreasing the value will lead to fewer clusters with more model profiles per cluster.

- *Minimum Correlation Percentile* – If there is repeat data selected to be from *Different time periods*, then this parameter specifies that any two model profiles assigned to the same cluster of profiles must have a correlation in their expression greater than the correlation of this percentile in the distribution of gene expression correlations between the repeats. For instance if this parameter value is 0.5, then any two model profiles assigned to the same cluster will have a correlation greater than the median correlation of gene expression correlations between the repeats. This parameter allows clustering of model profiles to be dependent on the noise in the data. If the *Minimum Correlation* parameter is set to -1, then only the *Minimum Correlation Percentile* parameter value will influence the clustering of model profiles. Similarly if the *Minimum Correlation Percentile* parameter is set to 0, then only the *Minimum Correlation* parameter value will influence the clustering of model profiles.

### 3.3.4 Gene Annotations Options

On the fourth panel, shown in Figure 9, a user may specify options related to gene annotations. The first three options allow one to filter annotations when the annotation file is in the official 15 column format. The last field, the *Category ID mapping file*, is useful in the case in which genes are annotated as belonging to a category outside the Gene Ontology. The options on this panel are as follows:

- *Only include annotations of type* {*Biological Process, Molecular Function, Cellular Component*} – These three checkboxes allow one to filter annotations that are not of the types checked. These three checkboxes only apply if the annotations are in the official 15 column GO format, in which case the annotation type is determined by the entry in the *Aspect* field (Column 9). An entry of $P$ in the *Aspect* field means the annotation is of type *Biological Process*, an entry of $F$ means the annotation is of type *Molecular Function*, and an entry of $C$ means the annotation is of type *Cellular Component*.

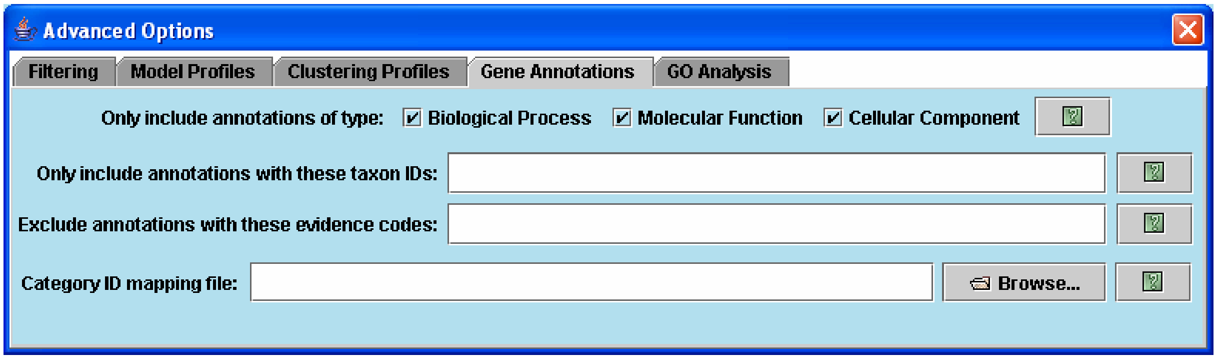


Figure 9: The above panel is used to specify options related to gene annotations.

- *Only include annotations with these taxon IDs* – Some annotation files contain annotations for multiple organism, and it might be desirable to use only annotations for certain organisms. To use only annotations for certain organisms enter the taxon IDs for the desired organisms delimited by either commas (','), semicolons (';'), or pipes ('|'). If this field is left empty, then any organism is assumed to be acceptable. More information about taxonomy codes and a search function to find the taxon code for an organism can be found at `http://www.ncbi.nlm.nih.gov/Taxonomy/`. Note that this parameter only applies when the annotations are in the official 15 column format. The taxonomy ID in the annotation file is in column 13 of the file, and the taxon IDs entered in this parameter field must match the entry in column 13 or match after prepending the string 'taxon:' to the ID. For example to use only annotations for a *Homo sapien* the string *9606* can be used.

- *Exclude annotations with these evidence codes* – This field takes a list of unacceptable evidence codes for gene annotations delimited by either a comma (','), semicolon (';'), or pipe ('|'). If this field is left empty, then all evidence codes are assumed to be acceptable. Evidence code symbols are *IEA, IC, IDA, IEP, IGI, IMP, IPI, ISS, RCA, NAS, ND, TAS, and NR*. Information about GO evidence codes can be found at `http://www.geneontology.org/GO.evidence.codes.shtml`. Note that this field only applies if the gene annotations are in the official 15 column GO annotation format. The evidence code is the entry in column 7. For example to exclude the annotations that were inferred from electronic annotation or a non-traceable author statement the field should contain *IEA;NAS*.

- *Category ID mapping file* – This file, which is optional, specifies a mapping between gene category IDs and category names for categories which are not official Gene Ontology categories. The mapping between IDs and names for official GO categories are defined in the file **gene_ontology.obo**. If a category ID appears in the gene annotation file, but does not correspond to an official Gene Ontology category and is not defined in a *Category ID mapping file*, then the category ID is used in place of the category name. A category ID mapping file has two columns delimited by a tab. The first column contains category IDs and the second column contains category names. Each line defines a mapping between one category ID and names. Below is a short sample file:

```
ID_A    CategoryNameA
ID_B    CategoryNameB
```

```
ID_C    CategoryNameC
```

#### 3.3.5 GO Analysis Options

The final advanced options panel, shown in Figure 10, controls options related to Gene Ontology (GO) enrichment

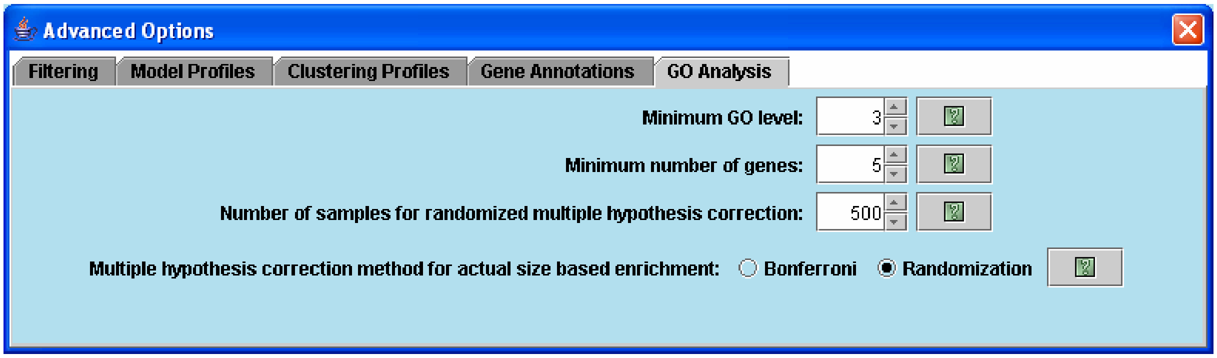


Figure 10: The above panel is used to specify options for the Gene Ontology enrichment analysis.

analysis. Note that categories that appear in a gene annotation file even if not part of the official Gene Ontology, are also included in a GO analysis. The parameters included on this panel are as follows:

- *Minimum GO level* – Any GO category whose level in the GO hierarchy is below this parameter will not be included in the GO analysis. The categories Biological Process, Molecular Function, and Cellular Component are defined to be at level 1 in the hierarchy. The level of any other term is the length of the longest path to one of these three GO terms in terms of the number of categories on the path. This parameter thus allows one to exclude the most general GO categories.

- *Minimum number of genes* – For a category to be listed in a gene enrichment analysis table, described in Section 5.2, the number of genes in the set being analyzed that also belong to the category must be greater than or equal to this parameter.

- *Number of samples for randomized multiple hypothesis correction* – This parameter specifies the number of random samples that should be made when computing multiple hypothesis corrected enrichment p-values by a randomization test. A randomization test is used when the p-value enrichment is based on the actual size of the set of genes and *Randomization* is selected next to the *Multiple hypothesis correction method for actual sized based enrichment* label. The Bonferroni correction is always used when the p-value enrichment is based on the expected size of the set of genes. The difference between actual and expected size enrichment is discussed in Section 4.3. Increasing this parameter will lead to more accurate corrected p-values for the randomization test, but will also lead to longer execution time to compute the values.

- *Multiple hypothesis correction method for actual sized based enrichment* – This parameter controls the correction method for actual size based GO enrichment. Expected size based p-values are always corrected using a Bonferroni correction. See Section 4.3 for a discussion on the differences between actual and expected size enrichment analysis. The parameter value can either be *Bonferroni* or *Randomization.* If *Bonferroni* is selected then a Bonferroni correction is applied where the uncorrected p-value is divided by the number

of categories meeting the minimum *Minimum GO level* and *Minimum number of genes* constraints. If *Randomization* is selected the corrected p-value is computed based on a randomization test where random samples of the same size of the set being analyzed is drawn. The number of samples is specified by the parameter *Number of samples for multiple hypothesis correction.* The corrected p-value for a p-value, $r$, is the proportion of random samples for which there is enrichment for any GO category with a p-value less than $r$. A Bonferroni correction is faster, but a randomization test leads to lower p-values.

# 4 Model Profiles Overview Interface

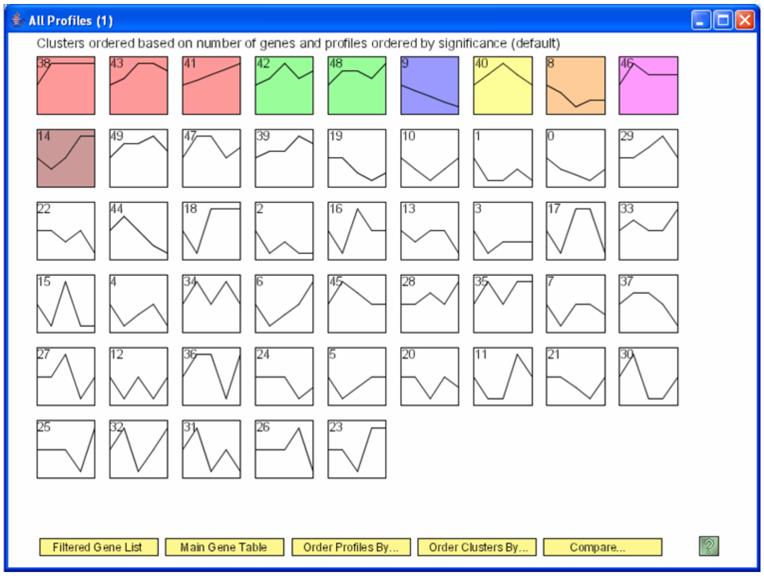


Figure 11: An example of the main profile overview interface. Each box corresponds to a model expression profile. Colored profiles have a statistically significant number of genes assigned. Clicking on a profile box display detailed information about the profile. The profiles and cluster of profiles can be reordered by various criteria by pressing *Order Profiles By* or *Order Clusters By*.

After the STEM clustering algorithm executes, the model profile overview interface appears. An example of such an interface is shown in Figure 11. Each box corresponds to a different model temporal expression profile. The number in the top left hand corner of a profile box is the model profile ID number. If the box is colored then a statistically significant number of genes were assigned to the model expression profile. Model profiles with the same color belong to the same cluster of profiles. Clicking on a model profile opens a new window that provides more detailed information about the model profile, and also the option to display gene tables and GO enrichment analysis tables. The window that appears with details about a model profile is discussed in depth in Section 5.

Along the bottom of the screen are several buttons: *Filtered Gene List*, *Main Gene Table*, *Order Profiles By*, *Order Clusters By*, *Compare*, and a help icon. The *Filtered Gene List* button displays a table of genes that were filtered, and thus not assigned to a model profile. The *Main Gene Table* button displays a table of genes that were not filtered, and thus assigned to a model profile. The *Order Profiles By* button opens a dialog window that allows one to reorder the model profiles on the main overview screen by a number of criteria. The *Order Clusters*

*By* button opens a dialog window that allows one to reorder the clusters of profiles, that is profiles are reordered with the constraint that profiles of the same color must be kept together. The main gene table, the filtered gene list, ordering profiles, and ordering clusters of profiles are explained in detail in the next four subsections. The *Compare* option which allows comparison with a data set from a different experimental condition is explained in Section 6. Pressing the help icon brings up the legend that appears in Figure 12 along with additional help information. The last subsection of this section, Section 4.5, describes how one can zoom in or out on any portion of the main window.

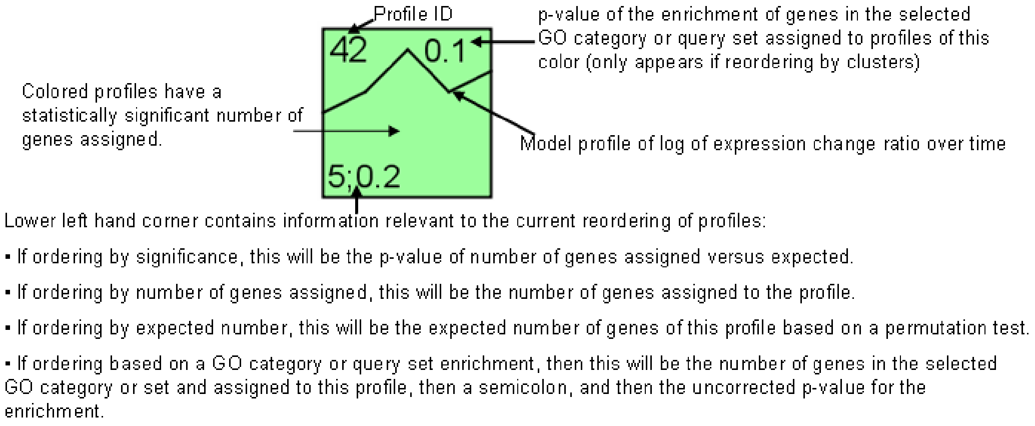


Figure 12: The legend that appears after pressing the help icon.

## 4.1 Main Gene Table

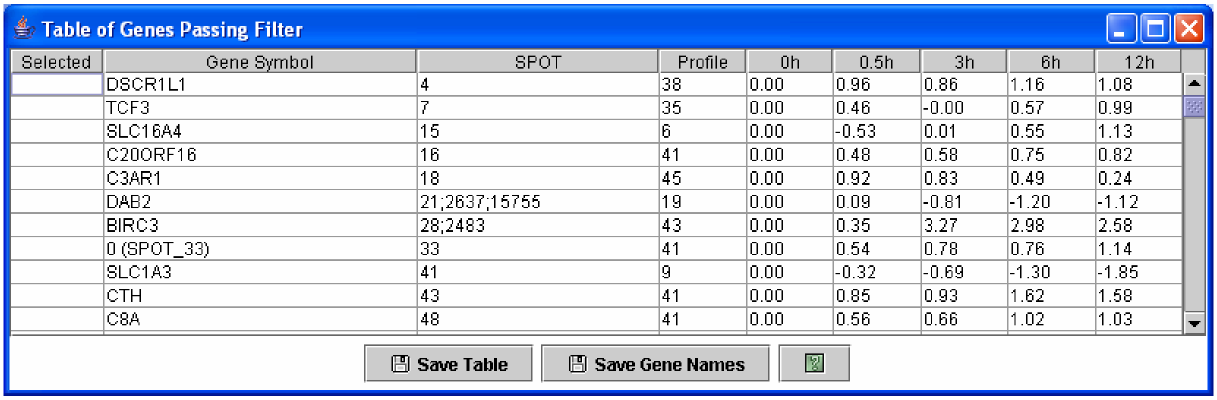

Table of Genes Passing Filter

| Selected | Gene Symbol | SPOT | Profile | 0h | 0.5h | 3h | 6h | 12h |
|---|---|---|---|---|---|---|---|---|
| | DSCR1L1 | 4 | 38 | 0.00 | 0.96 | 0.86 | 1.16 | 1.08 |
| | TCF3 | 7 | 35 | 0.00 | 0.46 | -0.00 | 0.57 | 0.99 |
| | SLC16A4 | 15 | 6 | 0.00 | -0.53 | 0.01 | 0.55 | 1.13 |
| | C20ORF16 | 16 | 41 | 0.00 | 0.48 | 0.58 | 0.75 | 0.82 |
| | C3AR1 | 18 | 45 | 0.00 | 0.92 | 0.83 | 0.49 | 0.24 |
| | DAB2 | 21;2637;15755 | 19 | 0.00 | 0.09 | -0.81 | -1.20 | -1.12 |
| | BIRC3 | 28;2483 | 43 | 0.00 | 0.35 | 3.27 | 2.98 | 2.58 |
| | 0 (SPOT_33) | 33 | 41 | 0.00 | 0.54 | 0.78 | 0.76 | 1.14 |
| | SLC1A3 | 41 | 9 | 0.00 | -0.32 | -0.69 | -1.30 | -1.85 |
| | CTH | 43 | 41 | 0.00 | 0.85 | 0.93 | 1.62 | 1.58 |
| | C8A | 48 | 41 | 0.00 | 0.56 | 0.66 | 1.02 | 1.03 |

Save Table | Save Gene Names

Figure 13: An example of a table that appears after pressing *Main Gene Table*. The table includes all genes that were not filtered, and thus assigned to a model profile.

Pressing the *Main Gene Table* button displays a table which has a row corresponding to every gene that was not filtered and thus assigned to a model profile. The table includes the gene's expression values after transformation and the profile(s) to which the gene was assigned. An example of such a table is shown in Figure 13. Clicking on a row of the table opens a new window containing detailed information about the profile to which the gene of the

row was assigned. This new window is described in Section 5. An option will also appear on the newly opened window to plot only the expression of the gene of the selected row.

The columns of the table are as follows:

- *Selected* – An entry in this column contains a 'Yes' if the gene of the row is part of a category or gene set by which the profiles are ordered, otherwise the field is empty.
- *Gene Symbol* – This column contains the gene symbols. The name for this column is read from the header in the data file.
- *Spot ID* – An entry in this column contains a list of spot IDs of spots which contain the gene of the row. The entries are delimited by a ';'. The header for this column is read from the data file if spot IDs are included in the data file.
- *Profile(s) Assigned* – The ID of the model profile, or in the case of a tie the profiles, for which the gene's expression pattern most closely matched and thus to which the gene was assigned.
- *Time Point columns* – The time series of gene expression levels for the gene after any selected transformation (*Log normalize data*, *Normalize data*, or *No normalization/add 0*). The header for these columns are read from the data file.

This table as all tables in STEM, can be sorted by any column. Click once on a column header to sort the table in ascending order by that column's values. Click twice on the column header to sort the table in descending order, and a third time to return the table to its original order. To cycle through the sorting options in the opposite order hold down the *Shift* button when clicking. To do a compound sort on multiple columns hold down the *Ctrl* button when clicking. Also as with all tables in STEM a user can save the contents of the table by pressing the *Save Table* button. As with any gene table in STEM, a user can also just save the list of gene names using the *Save Gene Names* button.

## 4.2 Filtered Gene List

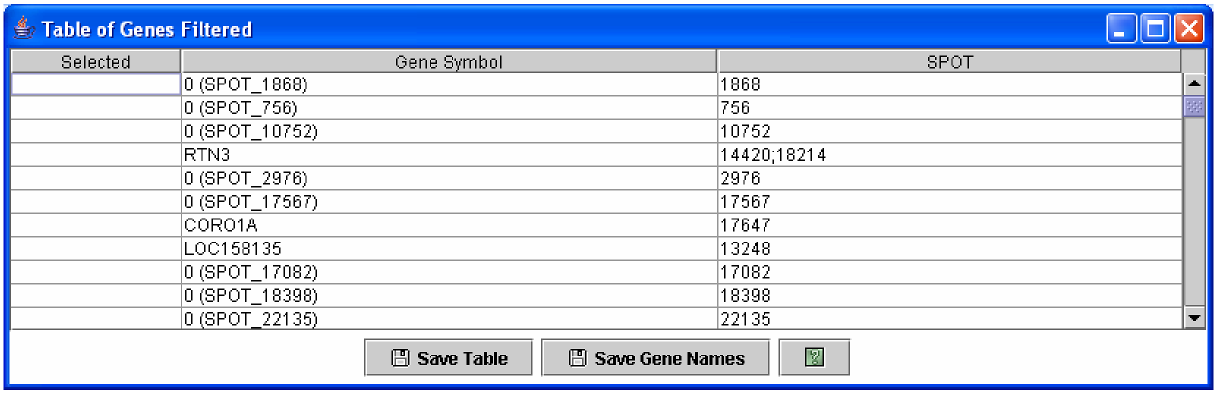


Figure 14: An example of a list of filtered genes

If a user presses the button *Filtered Gene List* a table such as the table in Figure 14 appears. The table contains a list of genes that were filtered and thus not assigned to a model expression profile. The parameters controlling filtering of genes are described in Section 3.3.1. The three columns of this table, the *Selected* column, the gene symbols column, and the spot ID column, are the same columns as the first three columns of the main gene table described in section 4.1.

## 4.3 Ordering Profiles

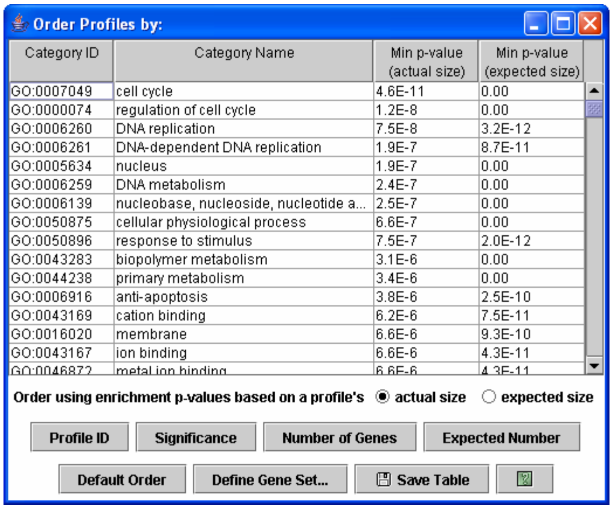


Figure 15: The dialog box through which the profiles can be reordered on the model profile overview interface. Clicking on a row of the table reorders the profiles by gene enrichment for genes of that category.

An important feature of STEM is its ability to easily reorder model profiles on the overview screen by a number criteria including the p-value of gene enrichment for any Gene Ontology category or a user defined gene set. To reorder the profiles, first press the button *Order Profiles By* on the model profile overview interface. A window such as the one in Figure 15 will then appear. The top portion of the window contains a table. The table contains an entry for every category containing at least one gene passing filter. The first two columns of this table are the category ID and category name. The third column contains the minimum p-value of the gene enrichment of genes for that category for any profile computed based on the profile's actual size. The fourth column also contains the minimum p-value of the gene enrichment of genes for that category for any profile, but computed based on a profile's expected size. The actual size of a profile is the number of genes assigned to the profile. The expected size is the number of genes expected to be assigned to the profile as computed based on a permutation test. During a permutation test the order of the time point values before transformation (*Log normalize data*, *Normalize data*, or *No normalization/add 0*) are randomly permuted, the transformation is applied, and then genes are assigned to model profiles. This is done for a large number of permutations, and the expected number of genes assigned to a profile is the average number of genes assigned over all permutations.

The actual size based p-value gene enrichment is computed based on a hypergeometric distribution. Suppose there are a total of $N$ genes on the microarray, $m$ of the these genes are in the category of interest, $v$ of the genes belong to the category of interest and were also assigned to the profile of the interest, and the number of gene's

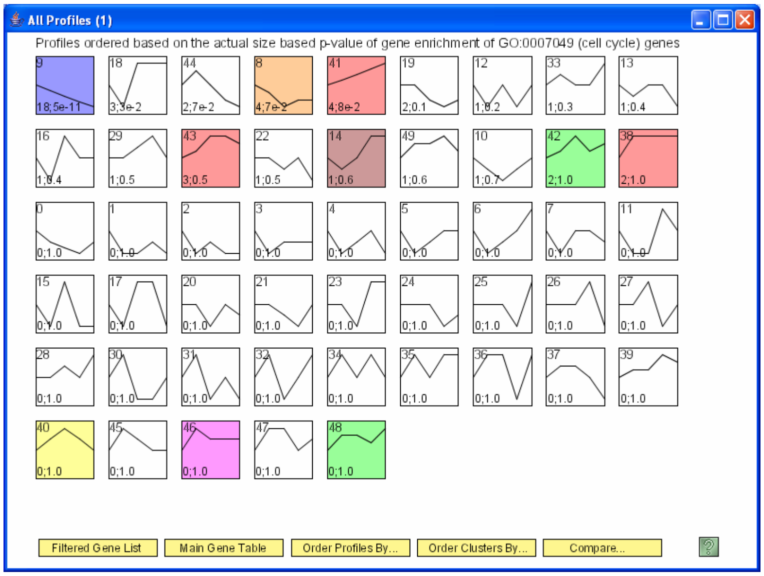


Figure 16: The main profile window with the profiles reordered by the actual size based p-value enrichment for cell cycle genes. The two numbers in the bottom left hand corner of each profile box are the number of cell cycle genes assigned to that profile, and then separated by a semicolon is the p-value of the gene enrichment for cell cycle genes in the profile.

assigned to the profile is $s_a$, then the p-value of seeing $v$ or more genes belonging to both the category of interest and assigned to the profile of interest can be computed as:

$$\sum_{i=v}^{\min(m,s_a)} \frac{\binom{m}{i}\binom{N-m}{s_a-i}}{\binom{N}{s_a}}$$

If the enrichment is computed based on a profile's expected size, $s_e$, then the p-value of seeing more than $v$ genes belonging to both the category and profile of interest can be computed based on a binomial distribution with parameters $m$ and $\frac{s_e}{N}$ as:

$$\sum_{i=v}^{m} \binom{m}{i} \left(\frac{s_e}{N}\right)^i \left(1-\frac{s_e}{N}\right)^{N-i}$$

If a profile has more genes assigned than expected, then it is possible a gene enrichment for a category will be significant under an expected size based enrichment while it is not significant under an actual size enrichment. Likewise if a profile has fewer genes assigned than expected, it is possible a gene enrichment for a category will be significant under an actual size based enrichment while it is not significant under an expected size based enrichment. If multiple independent processes happen to have the same temporal profile, then a significant gene enrichment for the process may be missed through an actual size enrichment, but detected through an expected size enrichment.

Clicking on a row of the table will reorder the profiles based on the p-value enrichment for the category of that row. Whether the p-value enrichment is computed based on the profile's *actual size* or *expected size* will depend on which is selected next to the label *Order using enrichment p-values based on a profile's*. Profiles are ordered row-wise from left to right and top to bottom based on the significance of the enrichment for the selected category.

The profile most enriched for the selected category appears in the top left corner. The next most enriched profile appears second in the top row and so on. For instance Figure 16 shows an example of the model profiles reordered based on an actual size enrichment for cell cycle genes. The numbers that appear in the bottom left hand corner of the model profile box are the number of genes assigned to the profile that also belong to the selected category and then separated by a semicolon the p-value enrichment.

Below the table are several buttons which give additional criteria to reorder profiles:

- *Profile ID* – Reorders profiles sequentially from left to right and top to bottom by their ID number, the number in the top left corner of the profile box (top left Figure 17). Profiles which go down initially will appear first, then profiles which hold steady initially, and then last will be profiles which go up initially.

- *Significance* – Reorders profiles based on the p-value significance of number of genes assigned to a profile being more than the number of genes expected (top right Figure 17). If $s_a$ genes were assigned to the profile and $s_e$ genes were expected and a total of $t$ genes passed filter, then the uncorrected p-value of seeing $s_a$ or more genes assigned to the profile is computed based on a binomial distribution with parameters $t$ and $\frac{s_e}{t}$. The p-value is computed as

$$\sum_{i=s_a}^{t} \binom{t}{i} \left(\frac{s_e}{t}\right)^i \left(1 - \frac{s_e}{t}\right)^{t-i}$$

  The most significant profiles appear to the left on the top row. When profiles are reordered based on this option, the profile significance p-value of a profile will appear in the bottom left-hand corner of its profile box.

- *Number of Genes* – Reorders profiles based on the number of genes assigned to the profile. The profiles with the most genes assigned appearing to the left on the top row (bottom left Figure 17). When profiles are reordered based on the number of genes assigned to the profile, the number of genes assigned to a profile appears in the bottom left-hand corner of its profile box.

- *Expected Number* – Reorders profiles based on the expected number of genes assigned to the profile. The expected number is computed based on a permutation test of the time points (bottom right Figure 17). The profiles with the greatest expected number genes assigned appear to the left on the top row. When profiles are reordered based on the expected number of genes assigned to the profile, the expected number of genes assigned to a profile appear in the bottom left-hand corner of its profile box.

- *Default Order* – Reorders the profile back to their original order. In the original default ordering all significant profiles appear before non-significant profiles. Profiles belonging to the same cluster are grouped together. Clusters are ordered based on the total number of genes assigned to any profile in the cluster. Within clusters of profiles and among non-significant profile, the profiles are ordered based on increasing p-value for the significance of the number of genes assigned versus what was expected.

- *Define Gene Set* – Pressing the *Define Gene Set* brings up a dialog box (Figure 18), which allows one to reorder profiles by enrichment for genes in a user defined gene set. Any gene which is checked will be included in the gene set. The button *Unselect All* unselects all genes in the set, while the button *Select All* selects all genes. A gene set can be loaded from a text file by pressing *Load Gene Set*, and then specifying

the name of the file containing the gene names. One gene name should appear on each line of the file, and there should be no header lines in a file. Pressing *Save Gene Set* exports the current selected genes to a text file. Pressing the button *Query Set* reorders the model profiles based on p-value gene enrichments for genes in the query set. As with GO categories the enrichment can be computed based on either the *actual size* or *expected size* of the profile depending upon which is selected in the *Order Profiles By* window. The set of genes can also be selected to be the set of genes assigned to a profile in comparison data set as is explained in Section 6.

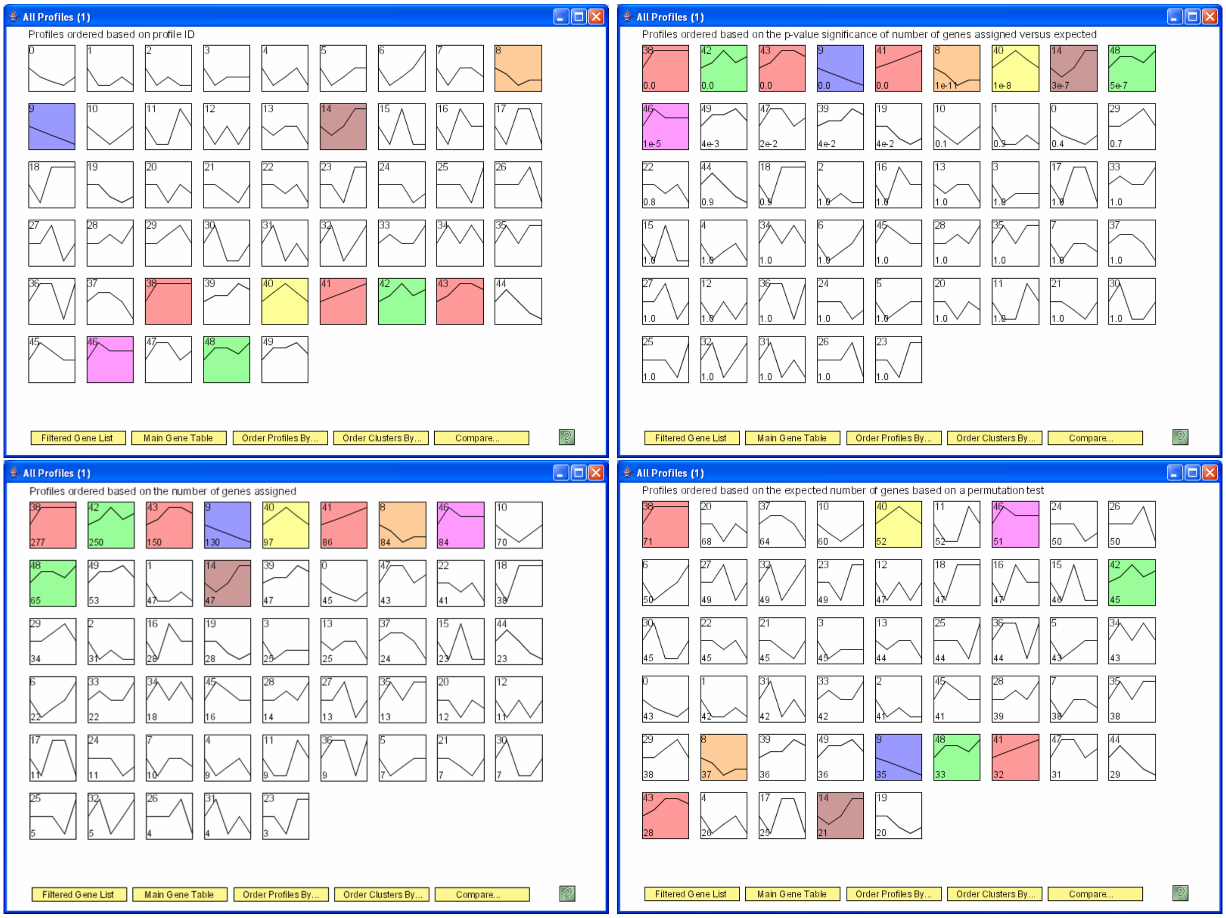


Figure 17: (Top left) Profiles are ordered by ID. (Top right) Profiles are ordered based on significance. (Bottom left) Profiles are ordered based on number of genes assigned. (Bottom right) Profiles are ordered based on the expected number of genes assigned.

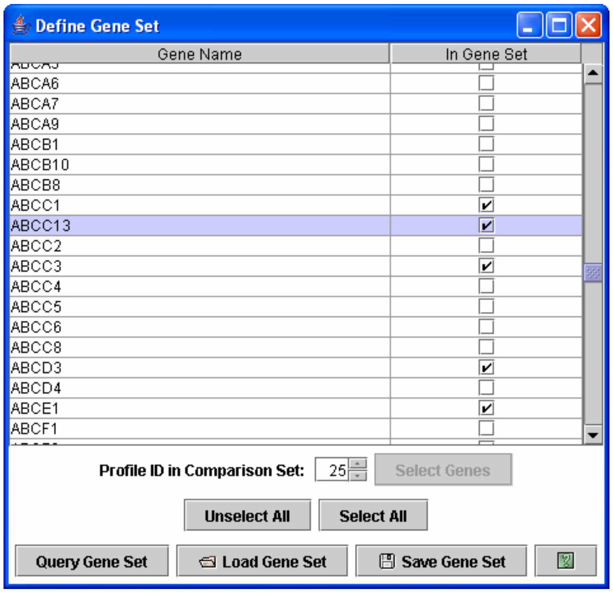


Figure 18: Dialog window through which to specify a user defined gene set. Genes which are checked are part of the set.

## 4.4 Ordering Clusters of Profiles

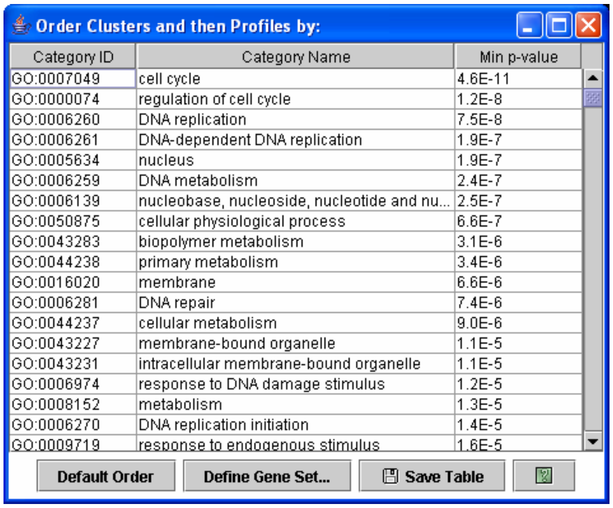


Figure 19: The dialog box through which the ordering of cluster of profiles can be changed

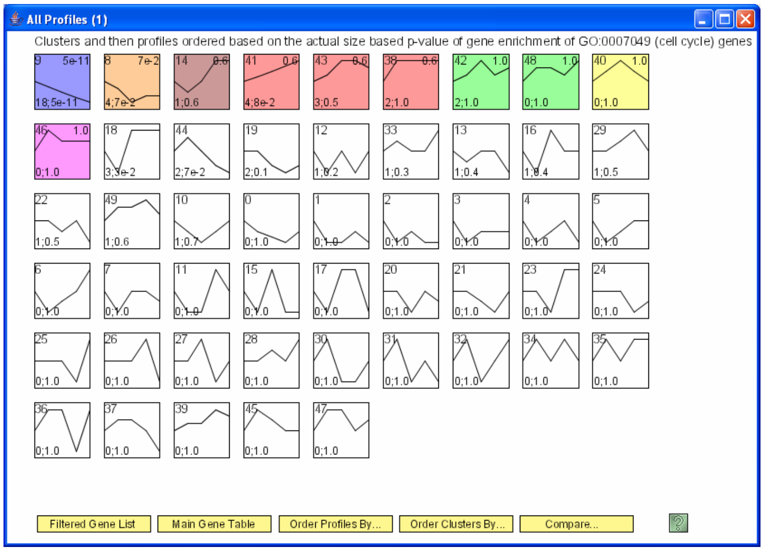


Figure 20: Cluster of profiles ordered based on enrichment for cell cycle genes.

When a user presses the *Order Clusters By* button on the main profile window a dialog box such as in Figure 19 appears. This window is a simplified version of the window that appears when a user presses *Order Profiles By*. Through this dialog box a user can reorder the cluster of profiles. A cluster of profiles is either a singleton profile or a group of profiles which all have a statistically significant number of genes assigned and are all similar to each other as defined based on parameters on the *Clustering panel* under *Advanced Options*. Profiles of the same cluster have the same color on the main profile window. When clusters of profiles are reordered, profiles of the same cluster are kept next to each other as appears in Figure 20. Also when reordering clusters of profiles, all non-statistically significant profiles are also reordered, but always appear after the cluster of statistically significant

profiles. As with reordering profiles discussed in the previous subsection, it is possible to reorder the cluster of profiles by gene enrichment for any category that appears in the table by clicking on the row of the category. The gene enrichment for a cluster of profiles is always computed based on the number of genes assigned to the cluster. As with reordering profiles one can reorder a cluster of profiles by a user defined gene set, by pressing the button *Define Gene Set* and then using a dialog such as appeared in Figure 18. The expected number of genes in a cluster being analyzed is not well defined, since the clusters of profiles are defined based on the data. When clusters of profiles are reordered based on a category or user defined gene set, the p-value enrichment for the cluster of profiles appears in the top right hand corner of the profile box of each profile that is part of the cluster. The number of genes of the category assigned to the profile and the p-value enrichment still appear in the lower left hand corner of the profile box. The *Default Order* button returns the profiles to their initial ordering as explained in Section 4.3.

### 4.5 Zooming and Panning

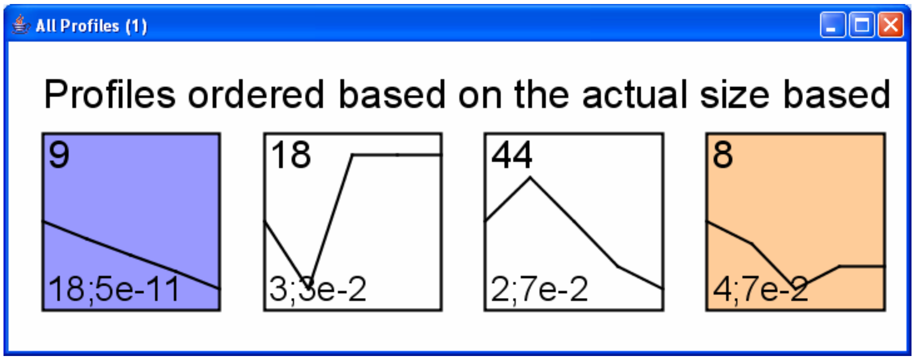


Figure 21: The above image shows a screen shot of the profile overview interface zoomed in on the four profiles most enriched for cell cycle.

As Figure 21 illustrates the model profile overview interface is zoomable. To zoom in, hold down the right mouse button and move the mouse to the right. To zoom out, hold down the right mouse button and move the mouse to the left. To pan hold down the left mouse button while not over a model profile box and then move the mouse in the desired direction. The ability to zoom in and out is powered by the the Piccolo Toolkit [1] which is distributed under a BSD license.

## 5 Model Profile Details Interface

When a user clicks on a model profile box on the model profile overview screen of the software, on a model profile box on the comparison interface screen discussed in Section 6, or on a row in the main gene table, a window with detailed information about the profile and the genes assigned to the profile appears in a new window (Figure 22). The window displays a graph of the expression values after transformation of all genes assigned to the profile. Along the top center of the window are two lines of text. The first line contains the model profile ID and a vector representing the expression pattern. The second line of the window contains a count of the number of

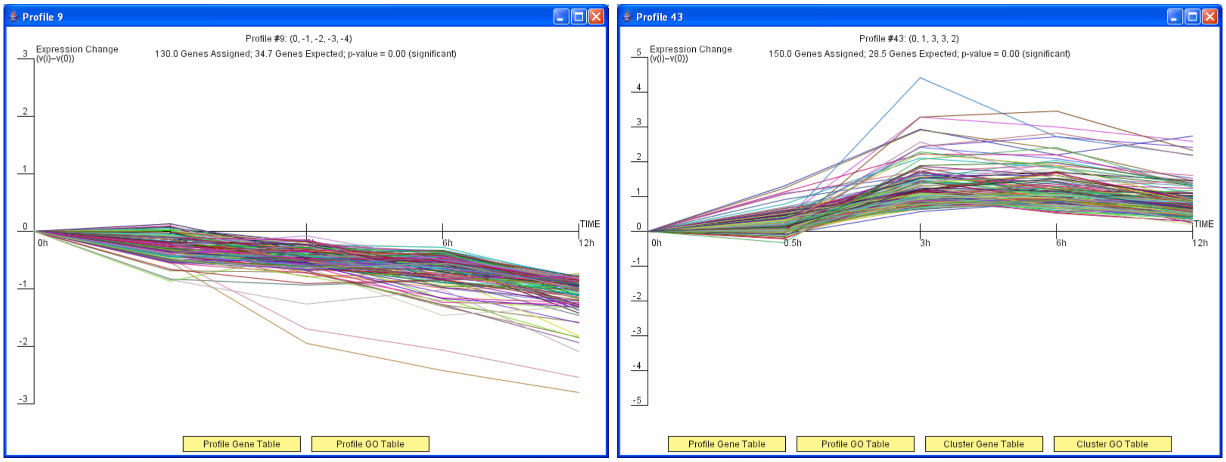


Figure 22: Example of detailed model profile information windows. The window plots a graph of all genes assigned to the profile. The text at top gives information about the profile including the number of genes assigned, the number of genes expected, and the p-value significance. The *Profile Gene Table* button displays a table of genes assigned to the profile, while the *Cluster Gene Table* displays a table of genes assigned to any profile in the profile's cluster of profiles. The *Profile GO Table* displays a gene category enrichment for genes assigned to the profile, while *Cluster GO Table* displays a gene category enrichment for genes assigned to any profile in the profile's cluster of profiles.

genes assigned to this model expression profile, a count of the expected number of genes assigned to the model profile based on a permutation test, the uncorrected p-value for the significance for the number of genes assigned being greater than the number expected, and whether or not this is statistically significant as defined by the parameters on the *Model Profiles* panel under *Advanced Options*. If the profiles are reordered by gene enrichment for genes belonging to a GO category or a user defined gene set, then an additional line of text will appear below the first two lines of text (Figures 23, 24). The additional line indicates the uncorrected p-value of the profile gene enrichment for the category or gene set the profiles are being ordered by. In parentheses are two ratios with a "vs." in between, thus having the form ($\frac{A}{B}$ vs. $\frac{C}{D}$). In first ratio, the numerator $A$, is the number of genes assigned to the profile that belong to the category or user defined gene set by which the profiles are ordered. The second number, $B$, is either the total number of genes assigned to the profile if the profiles are ordered based on *actual size* gene enrichment, or the expected number of genes assigned to the profile if the profiles are ordered based on *expected size* gene enrichment. In the second ratio the numerator, $C$, contains the total number of genes in the category or user defined gene set. The denominator in the second ratio, $D$, is the total number of genes on the array. The number to the right of the second ratio after the semicolon is the number of genes the profile is enriched for and is computed as $A - B \times \frac{C}{D}$. If the cluster of profiles are reordered based on a category or a user defined gene set, then an additional line appears below the profile enrichment line, about the category enrichment computed based on the set of genes assigned to any profile in the cluster.

Along the bottom of the window are several yellow buttons. Which buttons appear will depend upon how the profiles are ordered, through which interface the window was opened, and whether the profile is part of a non-singleton cluster of profiles. However every window will contain a *Profile Gene Table* and *Profile GO Table* button. The *Profile Gene Table* button displays a table with all the genes assigned to the profile. A gene table is described in Section 5.1. The profile *Profile GO Table* brings up a table with gene category enrichments among

genes assigned to the profile. A gene category enrichment table is described in Section 5.2. If a profile is part of cluster of profiles which is not a singleton, then two additional buttons will appear along the bottom row of the window, the *Cluster Gene Table* and *Cluster GO Table* buttons. The *Cluster Gene Table* button displays a gene table that includes all genes assigned to any profile in the cluster of profiles to which the profile belongs. The *Cluster GO Table* button displays a gene enrichment table that is based on enrichment for all genes assigned to any profile that is part of its cluster of profiles.

If the profiles or cluster of profiles are reordered based on a category, then two additional buttons will appear above the bottom row. Pressing the top of these two button will display a table of the genes that were assigned to the profile and also belong to the category by which the profiles are ordered. In Figure 23 this is the *Profile cell cycle Gene Table* button. Below this button is a button which gives the option to plot only the profile genes belonging to the category by which the profiles are ordered. This is the *Click to plot only profile cell cycle genes* button on the left side of Figure 23. Once this button is pressed, the button will be replaced with a button that says *Click to plot all profile genes* (right side of Figure 23), which gives the user the option to revert back to having all the profile genes plotted.

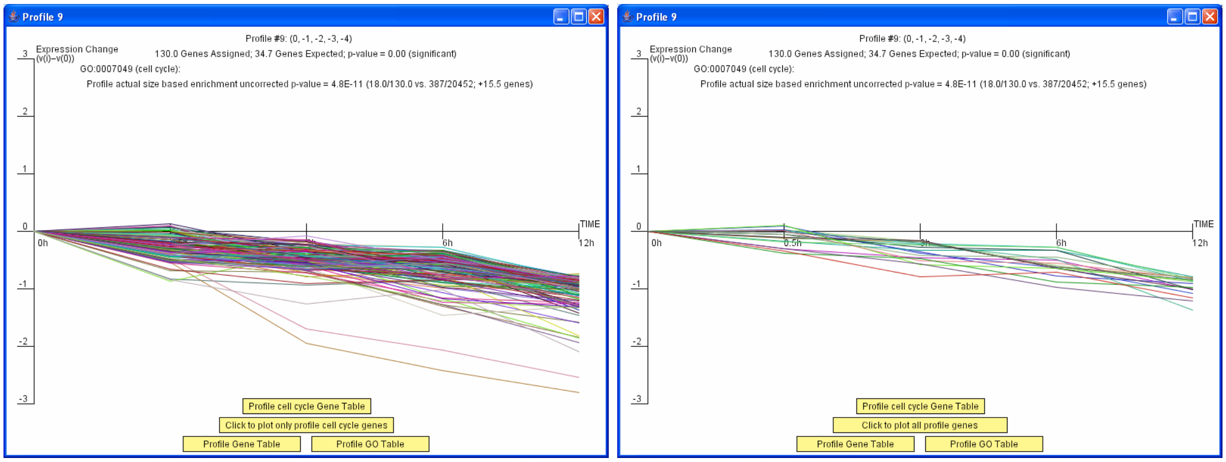


Figure 23: The window on left is an example of a model profile detailed information window that appears when the profiles are sorted based on enrichment for a GO category, in this case the cell cycle. The window on right is the same window after a user clicks *Click to plot only profile cell cycle genes*. Pressing the *Profile cell cycle Gene Table* displays a table of genes assigned to the profile that are also cell cycle genes.

If the profiles or cluster of profiles are ordered based on a user defined gene set, referred to as a query gene set, then there will be several additional buttons (Figure 24). The button *Click to plot only profile query set genes* replots the window with only profile genes that also belong to the user defined gene set. Pressing the button, will cause the button to be replaced with a *Click to plot all profile genes* button which pressing will revert to the original window. Above the *Profile Gene Table* and *Profile GO Table* are two buttons, the *Profile Query Gene Table* and the *Profile Query GO Table*. Pressing the *Profile Query Gene Table* displays a table with all genes assigned to the profile that also belong to the query gene set. Pressing the *Profile Query GO Table* displays a gene enrichment table for just the genes assigned to the profile that are also part of the query set. If the profile is part of a non-singleton cluster of profiles, then two additional buttons will appear, the *Cluster Query Gene Table* and *Cluster Query GO Table* buttons. These buttons are analogous to the *Profile Query Gene Table* and *Profile*

*Query GO Table* buttons, but are based on all genes in the query set that are assigned to any profile that is part of the profile's cluster of profiles.

If the profile window was opened by clicking on a row in the main gene table as described in Section 4.1, then a button will appear to plot only the gene of the row that was clicked on. This is the *Click to plot only gene STAM2* button on the left side of Figure 25. Once the button is pressed, the button will be replaced with the *Click to plot all profile genes* button (right side of Figure 25), which if pressed again will revert the window back to its original state.

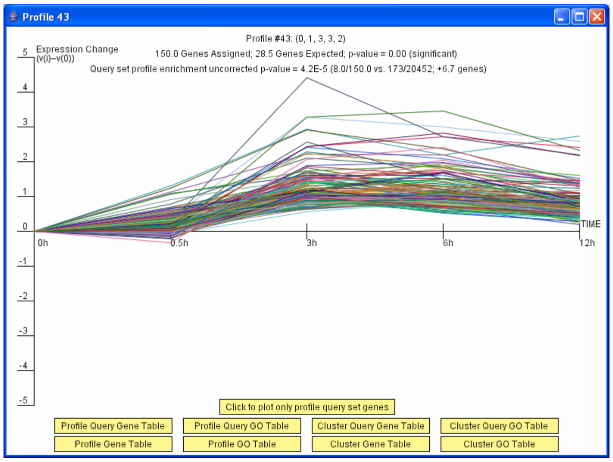


Figure 24: An example of a model profile detailed information window that appears when the profiles are ordered based on query set enrichment.

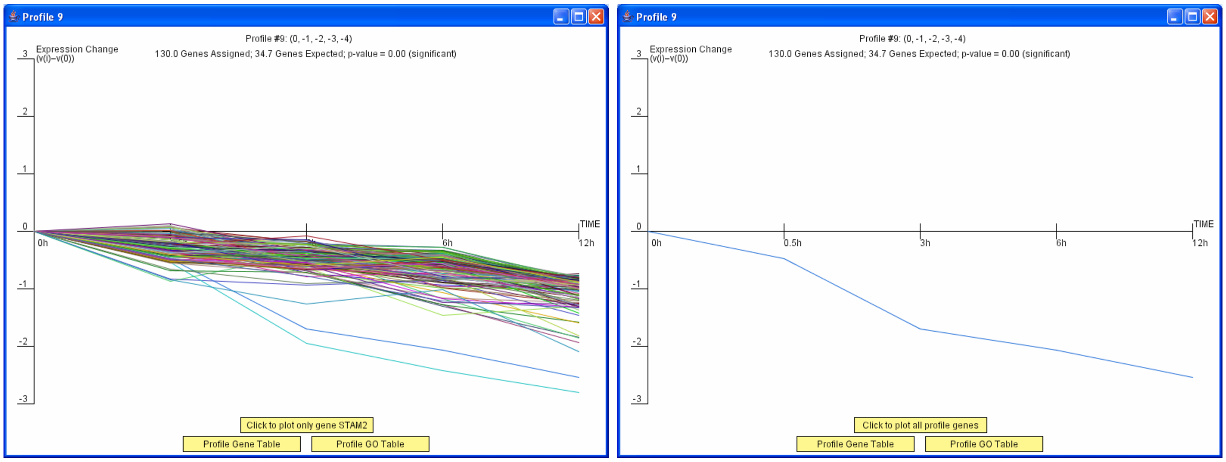


Figure 25: If the model profile window is opened from the main gene table, then there will be an option to plot just the gene selected. The image on the left initially appears, but pressing *Click to plot only gene STAM2* replots with only the gene STAM2.

## 5.1 Gene Table

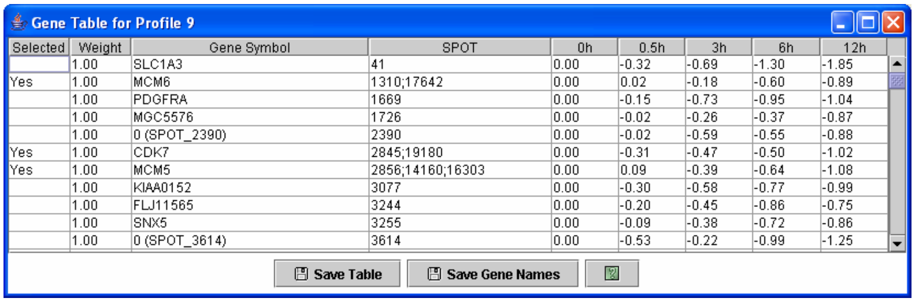

Gene Table for Profile 9

| Selected | Weight | Gene Symbol | SPOT | 0h | 0.5h | 3h | 6h | 12h |
|---|---|---|---|---|---|---|---|---|
| | 1.00 | SLC1A3 | 41 | 0.00 | -0.32 | -0.69 | -1.30 | -1.85 |
| Yes | 1.00 | MCM6 | 1310;17642 | 0.00 | 0.02 | -0.18 | -0.60 | -0.89 |
| | 1.00 | PDGFRA | 1669 | 0.00 | -0.15 | -0.73 | -0.95 | -1.04 |
| | 1.00 | MGC5576 | 1726 | 0.00 | -0.02 | -0.26 | -0.37 | -0.87 |
| | 1.00 | 0 (SPOT_2390) | 2390 | 0.00 | -0.02 | -0.59 | -0.55 | -0.88 |
| Yes | 1.00 | CDK7 | 2845;19180 | 0.00 | -0.31 | -0.47 | -0.50 | -1.02 |
| Yes | 1.00 | MCM5 | 2856;14160;16303 | 0.00 | 0.09 | -0.39 | -0.64 | -1.08 |
| | 1.00 | KIAA0152 | 3077 | 0.00 | -0.30 | -0.58 | -0.77 | -0.99 |
| | 1.00 | FLJ11565 | 3244 | 0.00 | -0.20 | -0.45 | -0.86 | -0.75 |
| | 1.00 | SNX5 | 3255 | 0.00 | -0.09 | -0.38 | -0.72 | -0.86 |
| | 1.00 | 0 (SPOT_3614) | 3614 | 0.00 | -0.53 | -0.22 | -0.99 | -1.25 |

Save Table    Save Gene Names

Figure 26: The above window is a gene table for a profile.

From a model profile details interface window, a user has the option to open or more gene tables such as the table that appears in Figure 26. As discussed in the beginning of the section, which genes appear in the table depends upon which button was pressed to open the table. A gene table has the following columns:

- *Selected* – This is the same column as in the main gene table. An entry in this column contains a 'Yes' if the gene of the row is part of a category or gene set by which the profiles are ordered, otherwise the field is empty.
- *Weight* – This field represents the weight of the assignment of the gene to the profile. If the profile the gene most closely matches is unique, then the value is one. If there is a tie as to which profile a gene most closely matches, then this value is one divided by the number of profiles a gene most closely matches.
- *Gene Symbol* – This column contains the gene symbols. The name for this column is read from the header in the data file.
- *Spot ID* – An entry in this column contains a list of spot IDs of spots which contain the gene of the row delimited by a ';'. The header for this column is read from the data file if spot IDs are included in the data file.
- *Time Point columns* – The time series of gene expression levels for the gene after any selected transformation (*Log normalize data*, *Normalize data*, or *No normalization/add 0*). The header for these columns are read from the data file.

As with all tables in STEM, this table can be sorted in ascending or descending order by any column by clicking on the column header. A user can also save the entire table using the *Save Table* button or just the gene names using the *Save Gene Names* button.

## 5.2 Gene Enrichment Analysis Table

From the window with details about a model profile a user has the option to display a table that includes gene enrichment for Gene Ontology (GO) categories along with any other categories that may appear in an annotation file. Figure 27 shows an example of such a table. As discussed at the beginning of the section the exact set of

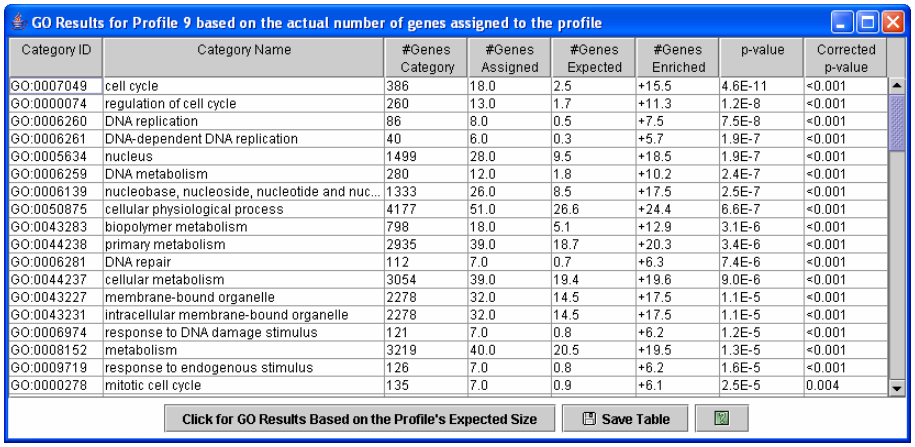

GO Results for Profile 9 based on the actual number of genes assigned to the profile

| Category ID | Category Name | #Genes Category | #Genes Assigned | #Genes Expected | #Genes Enriched | p-value | Corrected p-value |
|---|---|---|---|---|---|---|---|
| GO:0007049 | cell cycle | 386 | 18.0 | 2.5 | +15.5 | 4.6E-11 | <0.001 |
| GO:0000074 | regulation of cell cycle | 260 | 13.0 | 1.7 | +11.3 | 1.2E-8 | <0.001 |
| GO:0006260 | DNA replication | 86 | 8.0 | 0.5 | +7.5 | 7.5E-8 | <0.001 |
| GO:0006261 | DNA-dependent DNA replication | 40 | 6.0 | 0.3 | +5.7 | 1.9E-7 | <0.001 |
| GO:0005634 | nucleus | 1499 | 28.0 | 9.5 | +18.5 | 1.9E-7 | <0.001 |
| GO:0006259 | DNA metabolism | 280 | 12.0 | 1.8 | +10.2 | 2.4E-7 | <0.001 |
| GO:0006139 | nucleobase, nucleoside, nucleotide and nuc... | 1333 | 26.0 | 8.5 | +17.5 | 2.5E-7 | <0.001 |
| GO:0050875 | cellular physiological process | 4177 | 51.0 | 26.6 | +24.4 | 6.6E-7 | <0.001 |
| GO:0043283 | biopolymer metabolism | 798 | 18.0 | 5.1 | +12.9 | 3.1E-6 | <0.001 |
| GO:0044238 | primary metabolism | 2935 | 39.0 | 18.7 | +20.3 | 3.4E-6 | <0.001 |
| GO:0006281 | DNA repair | 112 | 7.0 | 0.7 | +6.3 | 7.4E-6 | <0.001 |
| GO:0044237 | cellular metabolism | 3054 | 39.0 | 19.4 | +19.6 | 9.0E-6 | <0.001 |
| GO:0043227 | membrane-bound organelle | 2278 | 32.0 | 14.5 | +17.5 | 1.1E-5 | <0.001 |
| GO:0043231 | intracellular membrane-bound organelle | 2278 | 32.0 | 14.5 | +17.5 | 1.1E-5 | <0.001 |
| GO:0006974 | response to DNA damage stimulus | 121 | 7.0 | 0.8 | +6.2 | 1.2E-5 | <0.001 |
| GO:0008152 | metabolism | 3219 | 40.0 | 20.5 | +19.5 | 1.3E-5 | <0.001 |
| GO:0009719 | response to endogenous stimulus | 126 | 7.0 | 0.8 | +6.2 | 1.6E-5 | <0.001 |
| GO:0000278 | mitotic cell cycle | 135 | 7.0 | 0.9 | +6.1 | 2.5E-5 | 0.004 |

Click for GO Results Based on the Profile's Expected Size | Save Table

Figure 27: A gene enrichment analysis table. Clicking on a row of the table brings up a gene table that includes only the genes annotated as belonging to the category of the row that are also in the set being analyzed. The above table is enrichment based on the actual size of the profile. Clicking on the button *Click for GO Results Based on the Profile's Expected Size* opens another table with GO results computed based on the expected size of a profile.

genes that the enrichment analysis is for depends upon which button was pressed to bring up the table. For a category to appear in the table, the number of genes in the set of genes being analyzed that belong to the category must be greater than or equal to the value of the *Minimum number of genes* parameter on the *GO Analysis* panel under *Advanced Options*. For official GO categories the level of the category must be greater than or equal to the value of the *Minimum GO level* parameter also on the *GO Analysis* panel under *Advanced Options*.

As discussed in Section 4.3 there are two ways to compute gene enrichment, one based on the actual size of the set and the other based on the expected size of the set. For clusters of profiles, gene enrichment is always based on the actual size of the set. For profiles, gene enrichment by default is based on the actual size of the set. However, there will be a button along the bottom of the window which says *Click for GO Result's Based on the Profile's Expected Size* that when pressed will open a new table where the enrichment analysis is based on the profile's expected size.

The columns of a gene enrichment table are as follows:

- *Category ID* – The ID for the category.
- *Category Name* – The name for the category.
- *# Genes Category* – The number of genes on the entire microarray that were annotated as belonging to the category.
- *# Genes Assigned* – The number of genes annotated as belonging to the category that are part of the set of genes being analyzed.
- *# Genes Expected* – The number of genes annotated as belonging to the category that were expected to be part of the set being analyzed. This value will depend on whether an actual size or expected size profile enrichment analysis is being conducted.
- *# Genes Enriched* – The difference between *# Genes Assigned* and *# Genes Expected*

- *p-value* – The uncorrected p-value of seeing this many or more genes from this category assigned to the set of genes being analyzed. This p-value will depend on whether an actual size or expected size enrichment analysis is being conducted. See Section 4.3 for a discussion on how the p-value is computed.

- *Corrected p-value* – The p-value corrected for testing a large number of GO categories. If the enrichment is based on a set's actual size and *Randomization* is selected as the value for *Multiple hypothesis correction method for actual size based enrichment* the corrected p-value is computed based on a randomization test. If the enrichment is computed based on a set's expected size or *Bonferroni* is selected as the value for *Multiple hypothesis correction method for actual size based enrichment*, then the corrected p-value is computed based on a Bonferroni correction. See section 3.3.5 for a discussion on these two methods for correcting GO enrichment p-values.

A gene enrichment table can be sorted by any column in ascending or descending order by clicking on the column header. The contents of the table can also be saved to a text file using the *Save Table* button. Clicking on a row of the gene enrichment table will display a gene table that only includes genes that belong to category of the row and also the set being analyzed. For example if a user clicked on the cell cycle row, a table such as that in Figure 28 will appear which contains only genes that were assigned to the profile being analyzed that were also annotated as being cell cycle genes.

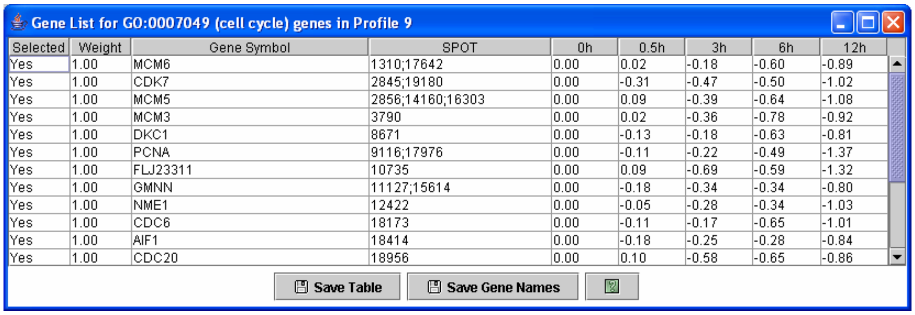


| Selected | Weight | Gene Symbol | SPOT | 0h | 0.5h | 3h | 6h | 12h |
|---|---|---|---|---|---|---|---|---|
| Yes | 1.00 | MCM6 | 1310;17642 | 0.00 | 0.02 | -0.18 | -0.60 | -0.89 |
| Yes | 1.00 | CDK7 | 2845;19180 | 0.00 | -0.31 | -0.47 | -0.50 | -1.02 |
| Yes | 1.00 | MCM5 | 2856;14160;16303 | 0.00 | 0.09 | -0.39 | -0.64 | -1.08 |
| Yes | 1.00 | MCM3 | 3790 | 0.00 | 0.02 | -0.36 | -0.78 | -0.92 |
| Yes | 1.00 | DKC1 | 8671 | 0.00 | -0.13 | -0.18 | -0.63 | -0.81 |
| Yes | 1.00 | PCNA | 9116;17976 | 0.00 | -0.11 | -0.22 | -0.49 | -1.37 |
| Yes | 1.00 | FLJ23311 | 10735 | 0.00 | 0.09 | -0.69 | -0.59 | -1.32 |
| Yes | 1.00 | GMNN | 11127;15614 | 0.00 | -0.18 | -0.34 | -0.34 | -0.80 |
| Yes | 1.00 | NME1 | 12422 | 0.00 | -0.05 | -0.28 | -0.34 | -1.03 |
| Yes | 1.00 | CDC6 | 18173 | 0.00 | -0.11 | -0.17 | -0.65 | -1.01 |
| Yes | 1.00 | AIF1 | 18414 | 0.00 | -0.18 | -0.25 | -0.28 | -0.84 |
| Yes | 1.00 | CDC20 | 18956 | 0.00 | 0.10 | -0.58 | -0.65 | -0.86 |

Figure 28: A table that appears after clicking on the cell cycle row in the gene enrichment table. The table only includes genes that were assigned to the profile being analyzed that were also annotated as being cell cycle genes.

# 6 Comparison

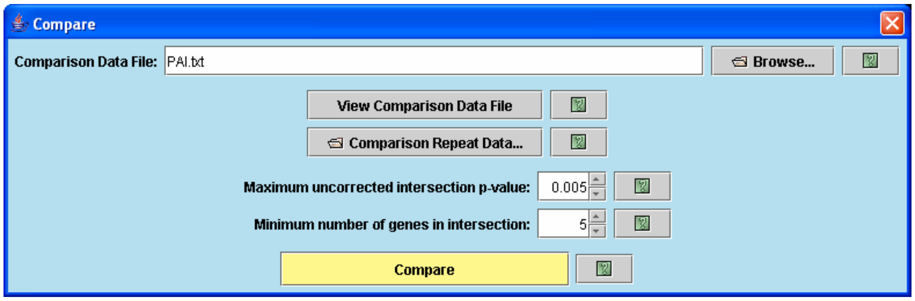


Figure 29: The comparison dialog box which is used to specify a comparison data set and parameters for gene set intersections of interest. Pressing the *Compare* opens two new windows, one is a model profile overview for the comparison data set, and the other is the main comparison window.

STEM facilitates the comparison of gene expression data sets from two different experimental conditions, and in particular allows automatic identification of statistically significant sets of genes which are co-expressed under both experimental conditions. STEM can automatically identify pairs of model profiles, one from each experiment, for which the intersection of the set genes assigned to the two profiles is statistically significant. Suppose there are $N$ genes on the microarray, $n_i$ genes are assigned to a profile $i$ in the first experiment, $n_j$ genes assigned to a profile $j$ in the second experiment, and a total of $t$ genes are in the intersection of the set of genes assigned to profile $i$ in the first experiment and profile $j$ in the second experiment, then the p-value of seeing $t$ are more genes in the intersection is computed based on the hypergeometric distribution to be

$$\sum_{m=t}^{\min(n_i,n_j)} \frac{\binom{n_j}{m}\binom{N-n_j}{n_i-m}}{\binom{N}{n_i}}$$

To specify a comparison data set from the model profile overview screen press the *Compare* button. Pressing this button will open a comparison dialog such as shown in Figure 29. A user can specify the name of comparison data file in the field *Comparison Data File*. Note that STEM requires that the comparison data have the same number of time points as the original data. Once a name of a file is specified, to view the contents of the file specified in the *Comparison Data File* press the button *View Comparison Data File*. Pressing the button *Comparison Repeat Data* will open a repeat dialog window from which to specify repeat data for the comparison experiment. This dialog window appears in Figure 4 and was described back in Section 3.1. Below the *Comparison Repeat Data* button are two parameters:

- *Maximum uncorrected intersection p-value* – The maximum uncorrected intersection p-value for the intersection to be of interest.
- *Minimum number of genes in intersection* – The minimum number of genes in the intersection of the set of genes assigned to two profiles for the intersection to be of interest.

Pressing the yellow *Compare* button will launch two new windows. One of the windows that is launched contains the model profile overview screen for the comparison data set. This is the same interface that is described in Section 4. The other window that appears is the main comparison window, an example of which is shown in

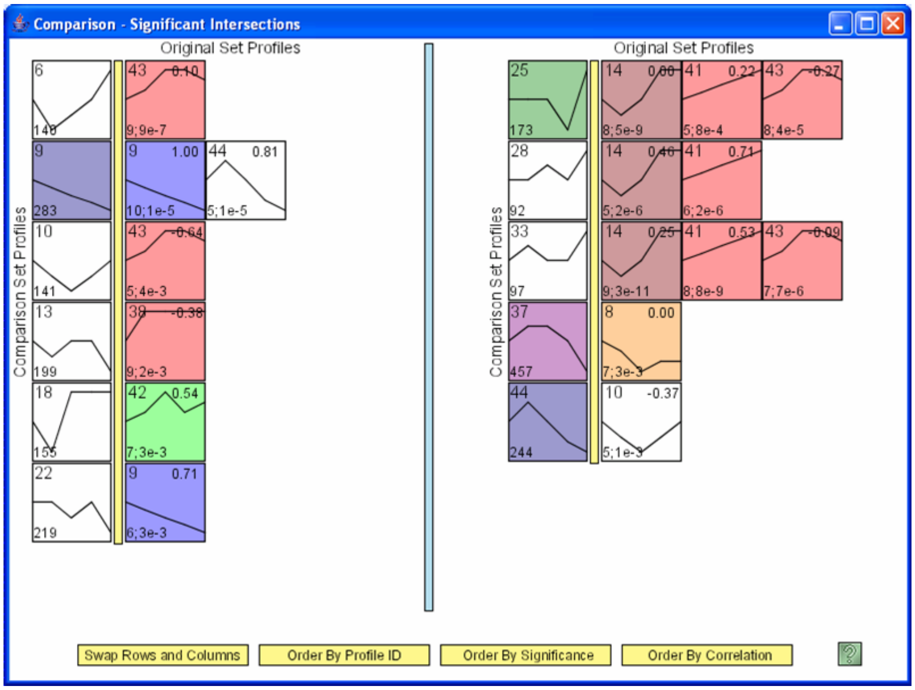


Figure 30: The main comparison window. If a profile appears to the right of the yellow bar, then a significant number of genes assigned to the profile were also assigned to the profile to the left of the yellow bar in the other experiment.

Figure 30. The window shows all profile pairs containing a gene intersection satisfying the size and p-value constraints specified on the comparison dialog. The interface layout has two halves with a blue bar separating the two halves; there is no significance associated with a profile appearing to the left or right of the blue bar. If the vertical text label on the left side of each half read "Original Set Profiles", then to the immediate left of the vertical yellow bar are profiles all of which are from the original data set. If the vertical label reads "Comparison Set Profiles", then the profiles to the left of the yellow bar are all from the comparison data set. To the right of the yellow bars are profiles from the other data set. If the profiles to the right of the yellow bar are from the comparison experiment, then the horizontal labels on the top of the screen will read "Comparison Set Profiles," while if the profiles to the right of the yellow bar are from the original experiment then the horizontal labels will read "Original Set Profiles." A profile appears to the right of the yellow bar if the intersection of the set genes assigned to it and the profile to the immediate left of the yellow bar satisfy the size and p-value constraints specified on the comparison dialog. The legend that appears when a user presses the help icon information appears in Figure 31 and explains what the various numbers mean on the profile boxes. This window as with the main profile screen is zoomable and pannable. Instructions for zooming and panning can be found in Section 4.5.

Clicking on a profile box to the right of a yellow bar launches a detail model profile window that includes the option to obtain information about the genes in the intersection between the profile clicked on and the profile to the immediate left of the yellow bar (left side Figure 32). Near the top of the window is a line of text indicating how many genes were in the intersection and the p-value of the intersection. The intersection profile window also contains a button which plots only those genes in the profile which were also assigned to the profile in its row to the left of the yellow bar in the other experiment. After pressing the *Click to plot only genes in intersection*

one has the option to press the button *Click to plot all profile genes* to revert back to the original screen. Two additional buttons that appear on the profile interface are the *Profile Intersect Gene Table* button and the *Profile Intersect GO Table* buttons. The *Profile Intersect Gene Table* button displays a gene table (Section 5.1) of genes assigned to this profile which were also assigned to the profile to the left of the yellow bar in the other experiment, that is the genes in the intersection. The *Profile Intersect GO Table* buttons displays a table (Section 5.2) with a gene enrichment analysis for genes in the intersection set. Clicking on a profile to the left of the yellow bar opens a window which displays information about the profile, but does not provide any information about gene intersections.

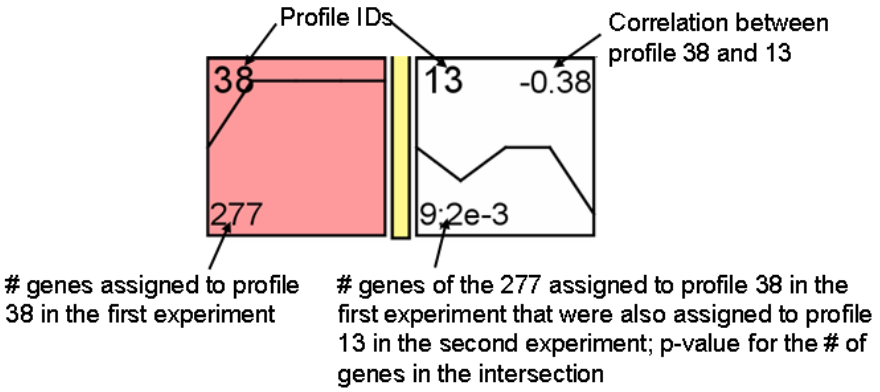


Figure 31: A legend for the comparison interface. The bottom left corner of profile boxes to the left of the yellow bar contain the number of genes assigned to the profile. The bottom left corner of profile boxes to the right of the yellow bar contains the number of genes assigned to the profile that were also assigned to the profile to the immediate left of the yellow bar, and then separated by a semicolon the p-value for seeing this many or more genes in the intersection. The upper right hand corner of the profile boxes to the right of the yellow bar contains the correlation with the profile to the left of the yellow bar.

On the bottom of the comparison window are four yellow buttons which are used to rearrange the profile boxes on the main window. These buttons function as follows:

- *Swap Rows and Columns* – Interchanges which data set is to the left of the yellow bar, and which is to the right of the yellow bar.
- *Order By Profile ID* – This button returns the profile pairs to their default ordering. By default the profiles to the left of the yellow are first ordered by increasing ID. Profiles to the right of the yellow bar are then ordered within the row by increasing ID.
- *Order By Significance* – This reorders profile pairs based on statistical significance of the gene set intersection. In any row, the profiles to the right of the yellow bar are ordered with increasing p-value for the gene set intersection with the profile to the left of the yellow bar. The profiles to the left of the yellow bar are ordered to have increasing minimum intersection p-value significance with a profile in its row to the right of the yellow bar.
- *Order By Correlation* – This reorders profile pairs based on correlation. In any row, the profiles to the right of the yellow bar are ordered based on increasing correlation with the profile to the left of the yellow bar.

The profiles to the left of the yellow bar are ordered to have increasing minimum correlation with a profile in its row to the right of the yellow bar.

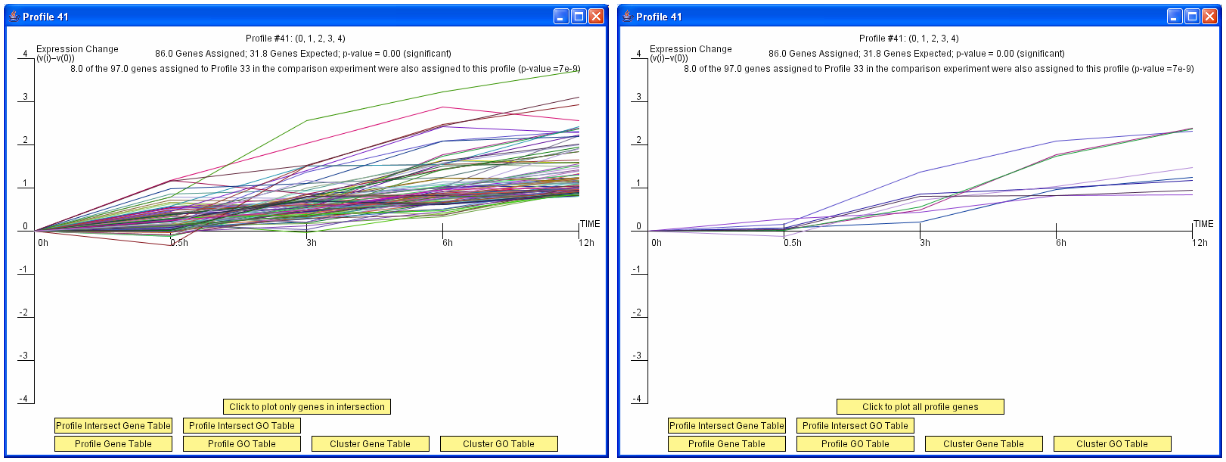


Figure 32: On the left is an example of a model profile window that appears when a model profile box to the right of a yellow bar is pressed. On the right is the same window after the button *Click to plot only genes in intersection* is pressed

As mentioned in Section 4.3 a user can reorder the profiles on the model profile overview screen based on gene enrichment for a user defined set. After the *Compare* button on the comparison dialog has been pressed, the user defined gene set can be defined based on sets of genes assigned to profile(s) in the other data set. This feature thus allows a user to visualize how a set of genes which all had the same expression profile(s) in one experiment responded in another experiment under different conditions. On the left of Figure 33 is the window to define a gene set by which to reorder the original data set model profiles, notice that the field *Profile ID in Comparison Set* is active. On the right of Figure 33 is the window to define a gene set by which to reorder the comparison data set model profiles, notice the field *Profile ID in Original Set* is active. Pressing the *Select* button selects those genes from the other experiment assigned to the profile of the ID displayed. Note that one can select genes from multiple profiles, since selecting an additional profile ID does not clear any currently selected genes. To create a gene set based on all the genes filtered in the other experiment set the profile ID value to "-1" and then press select genes.

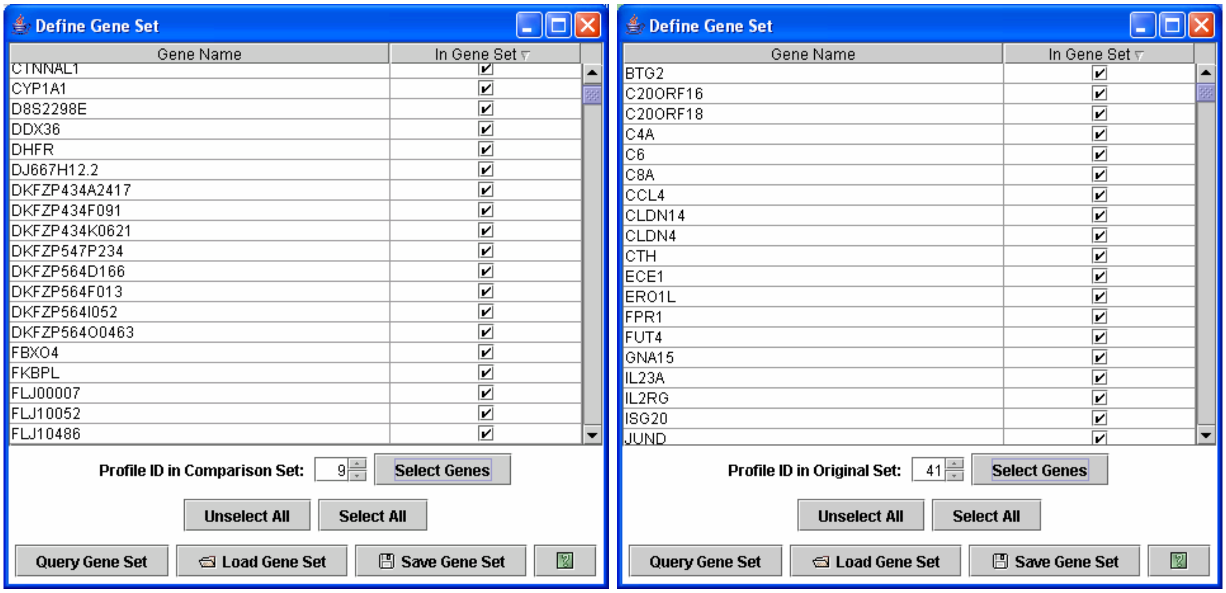


Figure 33: Dialog windows to define gene sets. The dialog window on the left is used to define a gene set to reorder model profiles from the original data set, while the dialog on the right is used to define a gene set to reorder model profiles from the comparison data set.

# 7 K-means

In addition to providing a novel clustering method designed for short time series expression data [3], STEM also provides an implementation of the standard $K$-means algorithm for clustering. To use the $K$-means clustering algorithm in STEM select *K-means* under *Clustering Method* (Figure 34). The $K$-means clustering algorithm partitions genes into $K$ sets, $S_1, S_2, ..., S_K$, where $K$ is an input parameter provided by a user in the field *Number of Clusters (K)*. Each set $S_i$ has a center $c_i$ associated with it where the center represents the mean of all genes assigned to the set $S_i$. After transformation described in Section 3.1 a gene $x_j$ and center $c_i$ are $T + 1$ element vectors that can be written as $(0, x_{j1}, x_{j2}, ..., x_{jT})$ and $(0, c_{i1}, c_{i2}, ..., c_{iT})$ respectively.

The $K$-means algorithm attempts tries to minimize the function

$$\sum_{i=1}^{K} \sum_{x_j \in S_i} \sum_{m=1}^{T} (x_{jm} - c_{im})^2$$

The $K$-means algorithm starts with randomly selected centers where in STEM's implementation the initial centers are chosen to be randomly selected genes. The algorithm then iterates between two steps until convergence. In one step each gene is reassigned to the cluster of the center to which it is closest. In the next step the center of each cluster is recomputed based on the new assignment of genes to clusters. The algorithm terminates when no changes in reassignment can be made. This algorithm is guaranteed to converge to a local minimum, but not a global minimum. The algorithm can be repeated for a number of different random starts with potentially a different clustering obtained from each start. Only the run with the best scoring final set of clusters is returned. The number of random starts is specified in the field *Number of Random Starts* on the main input interface. Increasing this parameter leads to a potentially slightly better clustering, at the expense of a slightly longer running time.

After the $K$-means algorithm executes the main output interface is displayed, an example of which appears in Figure 35. This interface is similar to the model profile overview interface described in Section 4 with a few differences of note. For $K$-means clustering each box on the interface corresponds to a cluster instead of a profile. The time series shown in the box is the average expression of all genes assigned to the cluster. The number in the top left hand corner of the box is a Cluster ID (see Figure 36 for a legend). All $K$-means cluster boxes appear white since no statistical significance is associate with them. The $K$-means cluster are by default ordered based on ID. IDs are assigned based on the cluster average expression value at the first time point. $K$-means cluster boxes can be reorder on the main interface analogous to the reordering of STEM profile boxes described in Section 4.3. Pressing the *Order Cluster By* button brings up the dialog box in Figure 37 through which the clusters can be reordered. The reordering criteria of the clusters can be the number of genes assigned to the cluster, or p-value enrichment for a GO category or user defined gene set.

Pressing a cluster box opens a window such as Figure 38 with detailed information about a $K$-means cluster similar to the model profile detailed interface described in Section 5. From this window one can open a table of all genes assigned to the cluster as one could do for all gene assigned to a STEM profile described in Section 5.1. Similarly one can open a table with GO analysis results for the set of genes assigned to the cluster as one could do for all genes assigned to a profile described in Section 5.2. The GO analysis can only be based on the actual size of the cluster since there is no notion of the expect sized of a $K$-means cluster.

Pressing the *Main Gene Table* on the main $K$-means interface is the same as described in Section 4.1 for the

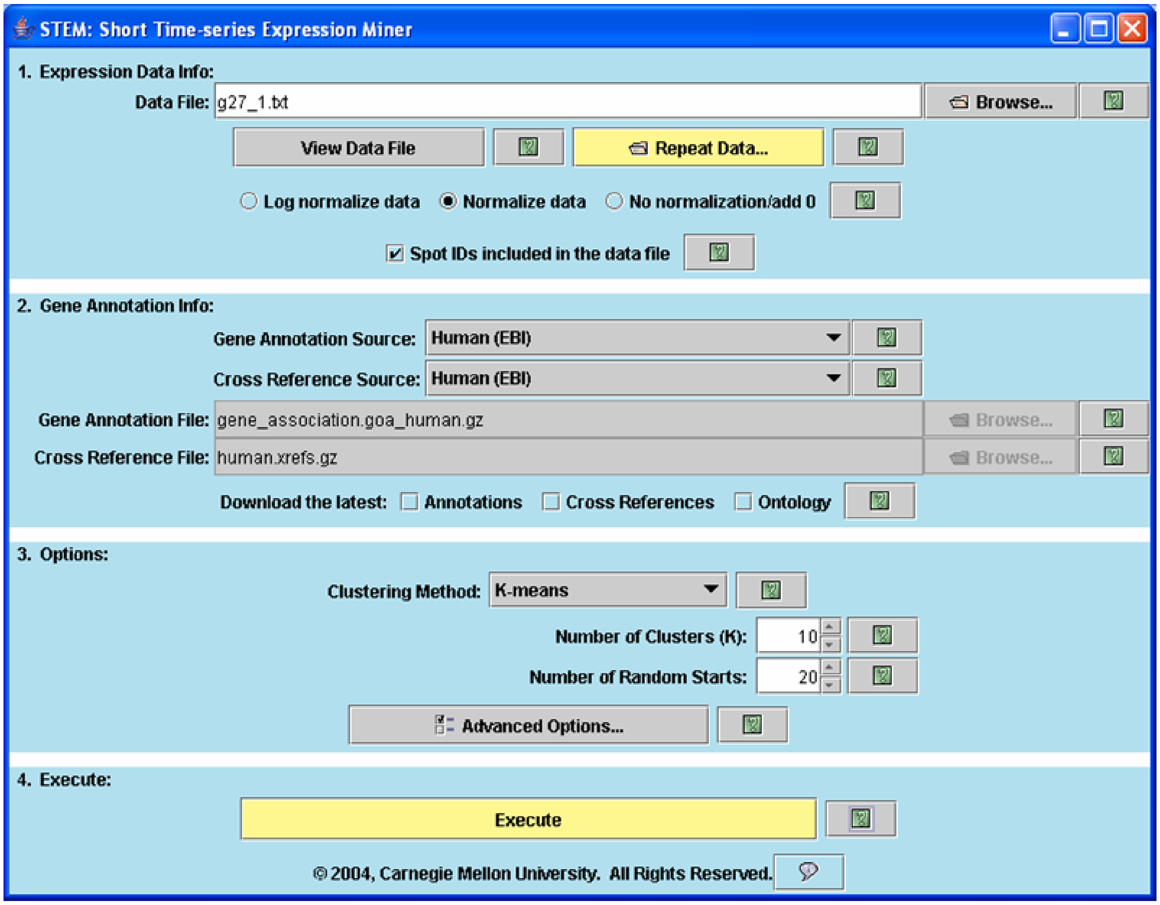


Figure 34: Above is the main input interface described previously in Section 3 with the clustering method set to $K$-means. Two parameters appear when $K$-means is selected that do not appear when the STEM clustering method is selected. These two parameters specify the number of clusters and the number of random starts.

STEM clustering method except the table has the cluster the gene was assigned to instead of the profile. The *Filter Gene Table* is identical to that described in Section 4.2. Comparison for $K$-means works the same way as described in Section 6 except STEM profiles are replaced with $K$-means clusters. Figure 39 shows the comparison legend for the comparison interface with $K$-means analogous to Figure 31 for comparison with STEM profiles.

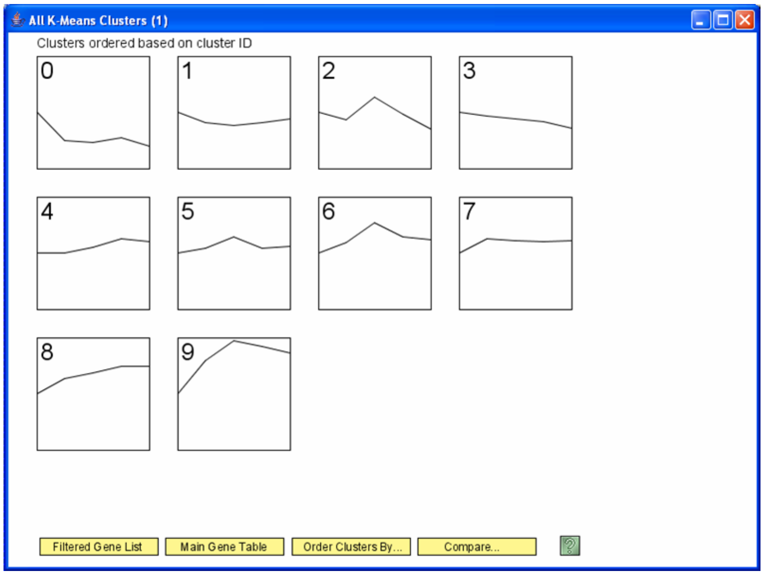


Figure 35: Above is the main output interface which is similar to the interface described in Section 4. Each box corresponds to a $K$-means cluster, and displays the average expression of genes in the cluster.

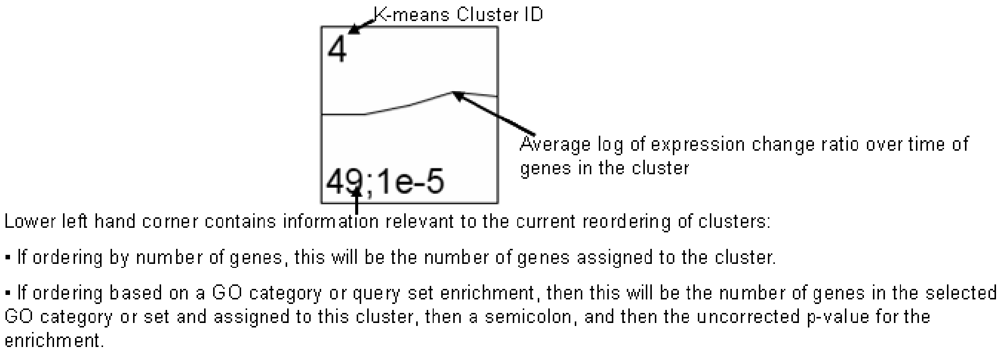


Figure 36: Legend for a $K$-means cluster box.

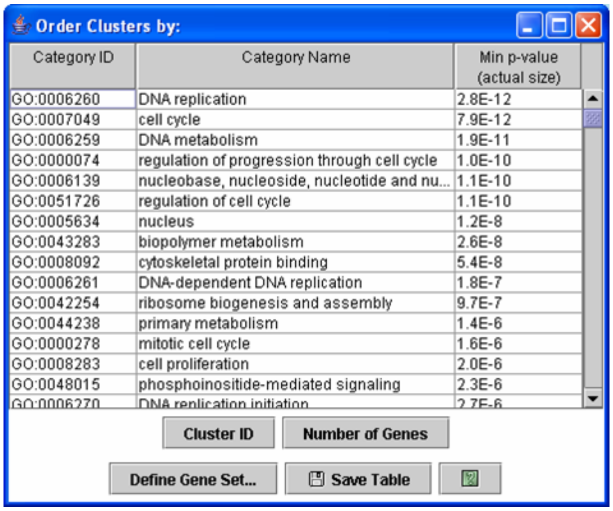


| Category ID | Category Name | Min p-value (actual size) |
|---|---|---|
| GO:0006260 | DNA replication | 2.8E-12 |
| GO:0007049 | cell cycle | 7.9E-12 |
| GO:0006259 | DNA metabolism | 1.9E-11 |
| GO:0000074 | regulation of progression through cell cycle | 1.0E-10 |
| GO:0006139 | nucleobase, nucleoside, nucleotide and nu... | 1.1E-10 |
| GO:0051726 | regulation of cell cycle | 1.1E-10 |
| GO:0005634 | nucleus | 1.2E-8 |
| GO:0043283 | biopolymer metabolism | 2.6E-8 |
| GO:0008092 | cytoskeletal protein binding | 5.4E-8 |
| GO:0006261 | DNA-dependent DNA replication | 1.8E-7 |
| GO:0042254 | ribosome biogenesis and assembly | 9.7E-7 |
| GO:0044238 | primary metabolism | 1.4E-6 |
| GO:0000278 | mitotic cell cycle | 1.6E-6 |
| GO:0008283 | cell proliferation | 2.0E-6 |
| GO:0048015 | phosphoinositide-mediated signaling | 2.3E-6 |
| GO:0006270 | DNA replication initiation | 2.7E-6 |

Figure 37: Above is the window to order $K$-means clusters. Clusters can be ordered based on ID, number of genes, or relevance to a GO Category or user defined gene set.

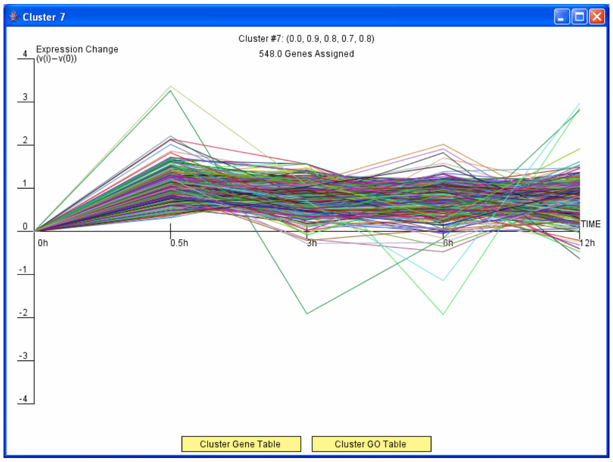


Figure 38: Above is an example of a window that provides detailed information about a $K$-means cluster.

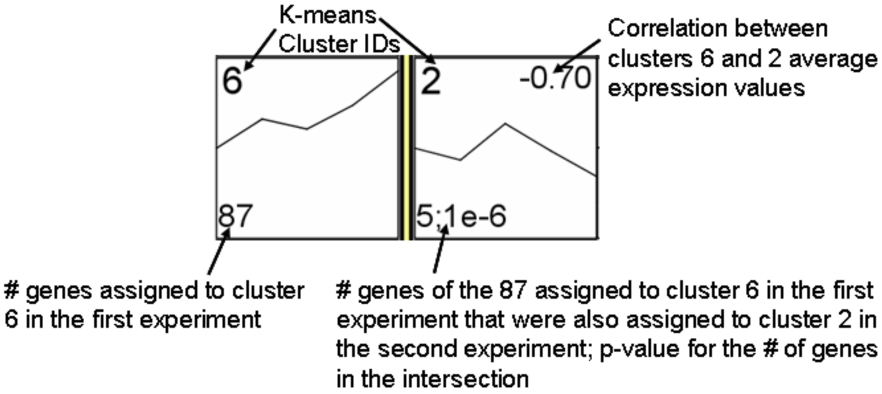


Figure 39: Legend for the comparison interface with $K$-means clusters.

# References

[1] Bederson B. B., Grosjean J., and Meyer J. Toolkit Design for Interactive Structured Graphics. *IEEE Transactions on Software Engineering*, 30 (8), pp. 535-546, 2004.

[2] Benjamini Y. and Hochberg Y. Controlling the False Discovery Rate: A Practical and Powerful Approach to Multiple Testing. *J. Roy Stat Soc*, B MET 57 (1): 289-300, 1995.

[3] Ernst J., Nau G., Bar-Joseph Z., Clustering Short Time-series Gene Expression Data. *Bioinformatics (Proceedings of ISMB 2005)*, 21 Suppl. 1, pp. i159-i168, 2005.

[4] Gene Ontology: tool for the unification of biology. The Gene Ontology Consortium. *Nature Genet.* 25: 25-29, 2000.

[5] Guillemin K., Salma N.R., Tompkins L.S., and Falkow S. Cag pathogenicity island-specific responses of gastric epithelial cells to Helicobacter pylori infection. *PNAS.* 99: 15136-15141, 2002.

# A Defaults File Format

As mentioned in the preliminary section the default settings for STEM can be specified in a file and used through the *-d* on the command line. Below is a sample file. The parameters names are on the left side and a tab separates them from their value. Lines which begin with a # are comments and are ignored.

```
#Main Input:
Data_File data.txt
Gene_Annotation_Source Human (EBI)
Gene_Annotation_File
Cross_Reference_Source Human (EBI)
Cross_Reference_File
Clustering_Method[STEM Clustering Method,K-means] STEM Clustering Method
Maximum_Number_of_Model_Profiles 50
Maximum_Unit_Change_in_Model_Profiles_between_Time_Points 2
Number_of_Clusters_K 10
Number_of_Random_Starts 20
Normalize_Data[Log normalize data,Normalize data,No normalization/add 0] Normalize data
Spot_IDs_included_in_the_data_file true

#Repeat Data:
Repeat_Data_Files(comma delimited list)
Repeat_Data_is_from[Different time periods,The same time period] Different time periods

#Comparison Data:
Comparison_Data_File compare.txt
Comparison_Maximum_Uncorrected_Intersection_pvalue    .005
Comparison_Minimum_Number_of_genes_in_intersection 5
Comparison_Repeat_Data_Files(comma delimited list)
Comparison_Repeat_Data_is_from[Different time periods,The same time period] Different time periods

#Filtering:
Maximum_Number_of_Missing_Values 0
Minimum_Absolute_Log_Ratio_Expression 1
Minimum_Correlation_between_Repeats 0
Pre-filtered_Gene_File

#Model Profiles:
Maximum_Correlation 1
Number_of_Permutations_per_Gene 50
Maximum_Number_of_Candidate_Model_Profiles 1000000
```

```
Significance_Level .05
Correction_Method[Bonferroni,False Discovery Rate,none] Bonferroni

#Clustering Profiles:
Clustering_Minimum_Correlation 0.7
Clustering_Minimum_Correlation_Percentile 0

#Gene Annotations:
Category_ID_file
Include_Biological_Process true
Include_Molecular_Function true
Include_Cellular_Process true
Only_include_annotations_with_these_evidence_codes
Only_include_annotations_with_these_taxon_IDs

#GO Analysis:
Minimum_GO_level 3
GO_Minimum_number_of_genes 5
Number_of_samples_for_randomized_multiple_hypothesis_correction 500
Multiple_hypothesis_correction_method_enrichment[Bonferroni,Randomization] Randomization
```

# B Using STEM for Standard Gene Ontology Enrichment Analysis

STEM may be used for standard Gene Ontology enrichment analysis for non-time series data in two ways. Given a data file of genes with a single time point column, STEM will perform a Gene Ontology enrichment analysis for those genes whose absolute value exceeds the value specified by the *Minimum Absolute Expression Change* parameter. In this case the base set of genes is all genes in the data file. STEM can also be used to do an enrichment analysis for an arbitrary set of genes and an arbitrary base set of genes. The set of genes to do an enrichment analysis on is specified in the *Data File* while the base set of genes are specified in the *Pre-filtered Gene File*. The first line of these files is a header line, and every line below the header line will contain one gene per line. As with a data file, the field *Spot IDs included in the data file* should be unchecked, unless spot IDs are the first column and gene symbols are the second column in which case the field should be checked. After pressing execute a gene enrichment analysis table will appear as described in Section 5.2.

# C Gene Annotation Sources

The table below lists all gene annotation data sets that can be selected under *Gene Annotation Source*. More information about these annotation data sets can be found here `http://www.geneontology.org/GO.current.annotations.shtml` and for the EBI annotations here `http://www.ebi.ac.uk/GOA/`. Subsets of the UniProt annotations for a large number of organisms provided by the European Bioninformatics Institute (EBI) can be found here `http://www.ebi.ac.uk/GOA/proteomes.html`, and can be used through the *User Provided* option under the *Gene Annotation Source*.

| **Annotation Set** | **Source** |
|---|---|
| Arabidopsis | European Bioinformatics Institute (EBI) |
| Arabidopsis thaliana | The Arabidopsis Information Resource (TAIR/TIGR) |
| Bacillus anthracis Ames | The Institute for Genomic Research (TIGR) |
| Caenorhabditis elegans | WormBase |
| Campylobacter jejuni RM1221 | The Institute for Genomic Research (TIGR) |
| Candida albicans | Candida Genome Database (CGD) |
| Chicken | European Bioinformatics Institute (EBI) |
| Cow | European Bioinformatics Institute (EBI) |
| Coxiella burnetii RSA 493 | The Institute for Genomic Research (TIGR) |
| Danio rerio | The Zebrafish Information Network (ZFIN) |
| Dehalococcoides ethenogenes 195 | The Institute for Genomic Research (TIGR) |
| Dictyostelium discoideum | DictyBase |
| Drosophila melanogaster | FlyBase |
| Geobacter sulfurreducens PCA | The Institute for Genomic Research (TIGR) |
| Human | European Bioinformatics Institute (EBI) |
| Leishmania major | Sanger GeneDB |
| Listeria monocytogenes 4b F2365 | The Institute for Genomic Research (TIGR) |
| Methylococcus capsulatus Bath | The Institute for Genomic Research (TIGR) |
| Mouse | European Bioinformatics Institute (EBI) |
| Mus musculus | Mouse Genome Informatics (MGI) |
| Oryza sativa | Gramene |
| PDB | European Bioinformatics Institute (EBI) |
| Plasmodium falciparum | Sanger GeneDB |
| Pseudomonas syringae DC3000 | The Institute for Genomic Research (TIGR) |
| Rat | European Bioinformatics Institute (EBI) |
| Rattus norvegicus | Rat Genome Database (RGD) |
| Saccharomyces cerevisiae | Saccharomyces Genome Database (SGD) |
| Schizosaccharomyces pombe | Sanger GeneDB |
| Shewanella oneidensis MR-1 | The Institute for Genomic Research (TIGR) |
| Silicibacter pomeroyi DSS-3 | The Institute for Genomic Research (TIGR) |
| Trypanosoma brucei | Sanger GeneDB |
| Trypanosoma brucei chr 2 | The Institute for Genomic Research (TIGR) |
| UniProt | European Bioinformatics Institute (EBI) |
| Vibrio cholerae | The Institute for Genomic Research (TIGR) |
| Zebrafish | European Bioinformatics Institute (EBI) |
